# Extraction and analysis of signatures from the Gene Expression Omnibus by the crowd

**DOI:** 10.1038/ncomms12846

**Published:** 2016-09-26

**Authors:** Zichen Wang, Caroline D. Monteiro, Kathleen M. Jagodnik, Nicolas F. Fernandez, Gregory W. Gundersen, Andrew D. Rouillard, Sherry L. Jenkins, Axel S. Feldmann, Kevin S. Hu, Michael G. McDermott, Qiaonan Duan, Neil R. Clark, Matthew R. Jones, Yan Kou, Troy Goff, Holly Woodland, Fabio M R. Amaral, Gregory L. Szeto, Oliver Fuchs, Sophia M. Schüssler-Fiorenza Rose, Shvetank Sharma, Uwe Schwartz, Xabier Bengoetxea Bausela, Maciej Szymkiewicz, Vasileios Maroulis, Anton Salykin, Carolina M. Barra, Candice D. Kruth, Nicholas J. Bongio, Vaibhav Mathur, Radmila D Todoric, Udi E. Rubin, Apostolos Malatras, Carl T. Fulp, John A. Galindo, Ruta Motiejunaite, Christoph Jüschke, Philip C. Dishuck, Katharina Lahl, Mohieddin Jafari, Sara Aibar, Apostolos Zaravinos, Linda H. Steenhuizen, Lindsey R. Allison, Pablo Gamallo, Fernando de Andres Segura, Tyler Dae Devlin, Vicente Pérez-García, Avi Ma'ayan

**Affiliations:** 1 Department of Pharmacological Sciences, BD2K-LINCS Data Coordination and Integration Center, Illuminating the Druggable Genome Knowledge Management Center, Icahn School of Medicine at Mount Sinai, One Gustave L. Levy Place Box 1215, New York, New York 10029, USA; 2 Fluid Physics and Transport Processes Branch, NASA Glenn Research Center, 21000 Brookpark Rd, Cleveland, Ohio 44135, USA; 3 Center for Space Medicine, Baylor College of Medicine, 1 Baylor Plaza, Houston, Texas 77030, USA; 4 Daylesford, the Fairway, Weybridge, Surrey KT13 0RZ, UK; 5 School of Biosciences, University of Nottingham, Sutton Bonington Campus, Sutton Bonington, Leicestershire LE12 5RD, UK; 6 Department of Biological Engineering, Massachusetts Institute of Technology, Cambridge, Massachusetts 02139, USA; 7 David H. Koch Institute for Integrative Cancer Research, Massachusetts Institute of Technology, Cambridge, Massachusetts 02139, USA; 8 Department of Materials Science & Engineering, Massachusetts Institute of Technology, Cambridge, Massachusetts 02139, USA; 9 The Ragon Institute of MGH, MIT, and Harvard, 400 Technology Square, Cambridge, Massachusetts 02139, USA; 10 Paediatric Allergology and Pulmonology, Dr von Hauner University Children's Hospital, Ludwig-Maximilians-University of Munich, Member of the German Centre for Lung Research (DZL), Lindwurmstrasse 4, Munich 80337, Germany; 11 Spinal Cord Injury Service, Veteran Affairs Palo Alto Health Care System, Palo Alto, California 94304, USA; 12 Department of Neurosurgery, Stanford School of Medicine, Stanford, California 94304, USA; 13 Department of Research, Institute of Liver & Biliary Sciences, D1, Vasant Kunj, New Delhi 110070, India; 14 Department of Biochemistry III, University of Regensburg, Universitätsstrasse 31, Regensburg 93053, Germany; 15 Department of Pharmacology and Toxicology, University of Navarra, Pamplona, Irunlarrea 1, Pamplona 31008, Spain; 16 Warsaw School of Information Technology under the auspices of the Polish Academy of Sciences, 6 Newelska St, Warsaw 01–447, Poland; 17 Plomariou 1 St, 15126 Athens, Greece; 18 Department of Biology, Faculty of Medicine, Masaryk University, Brno 625 00, Czech Republic; 19 IMIM-Hospital Del Mar, PRBB Barcelona, Dr Aiguader, Barcelona 88.08003, Spain; 20 85 Hailey Ln, Apt C-11, Strasburg, Virginia 22657, USA; 21 Department of Biology, Shenandoah University, 1460 University Dr Winchester, Winchester, Virginia 22601, USA; 22 IBM India Pvt Ltd., Bengaluru 560045, India; 23 Dr Aleksandra Sijacica 20, Backa Topola 24300, Serbia; 24 Department of Biological Sciences, 600 Fairchild Center, Mail Code 2402, Columbia University, New York, New York 10032, USA; 25 Center for Research in Myology, Sorbonne Universités, UPMC Univ Paris 06, INSERM UMRS975, CNRS FRE3617, 47 Boulevard de l'hôpital, Paris 75013, France; 26 13-1, Higashi 4-chome Shibuya-ku, Tokyo 150-0011, Japan; 27 Department of Biology and Institute of Genetics, Universidad Nacional de Colombia, Bogota, Cr. 30 # 45-08, Colombia; 28 Center for Interdisciplinary Cardiovascular Sciences, Brigham and Women's Hospital, 3 Blackfan Circle, Boston, Massachusetts 02115, USA; 29 Department of Human Genetics, Faculty of Medicine and Health Sciences, University of Oldenburg, Ammerländer Heerstrasse 114-118, Oldenburg 26129, Germany; 30 2312 40th ST NW #2, Washington DC 20007, USA; 31 Technical University of Denmark, National Veterinary Institute, Bülowsvej 27 Building 2-3, Frederiksberg C 1870, Denmark; 32 Protein Chemistry and Proteomics Unit, Biotechnology Research Center, Pasteur Institute of Iran, No. 358, 12th Farwardin Ave, Jomhhoori St, Tehran 13164, Iran; 33 School of Biological Sciences, Institute for Researches in Fundamental Sciences, Niavaran Square, P.O.Box, Tehran 19395-5746, Iran; 34 University of Salamanca, Salamanca, Madrid 37008, Spain; 35 Division of Clinical Immunology, Department of Laboratory Medicine, Karolinska Institute, Alfred Nobels Allé 8, level 7, Stockholm SE141 86, Sweden; 36 Department of Life Sciences, School of Sciences, European University Cyprus, 6 Diogenes Str. Engomi, P.O.Box 22006, Nicosia 1516, Cyprus; 37 Anna Blamansingel 216, Amsterdam 102 SW, Netherlands; 38 7300 Brompton #6024, Houston, Texas 77025, USA; 39 Aligustre 30 1-C, Madrid 28039, Spain; 40 CICAB, Clinical Research Centre, Extremadura University Hospital, Elvas Av., s/n. 06006 Badajoz 06006, Spain; 41 69 Brown Street, Box 8278, Providence, Rhode Island 02912, USA; 42 Consejo Superior de Investigaciones Científicas, Centro Nacional de Biotecnología, Department of Immunology and Oncology, c/Darwin, 3 Madrid 28049, Spain

## Abstract

Gene expression data are accumulating exponentially in public repositories. Reanalysis and integration of themed collections from these studies may provide new insights, but requires further human curation. Here we report a crowdsourcing project to annotate and reanalyse a large number of gene expression profiles from Gene Expression Omnibus (GEO). Through a massive open online course on Coursera, over 70 participants from over 25 countries identify and annotate 2,460 single-gene perturbation signatures, 839 disease versus normal signatures, and 906 drug perturbation signatures. All these signatures are unique and are manually validated for quality. Global analysis of these signatures confirms known associations and identifies novel associations between genes, diseases and drugs. The manually curated signatures are used as a training set to develop classifiers for extracting similar signatures from the entire GEO repository. We develop a web portal to serve these signatures for query, download and visualization.

## Abstract (web summary)

A wealth of gene expression data is publicly available, yet is little use without additional human curation. Ma'ayan and colleagues report a crowdsourcing project involving over 70 participants to annotate and analyse thousands of human disease-related gene expression datasets.

## Graphical abstract

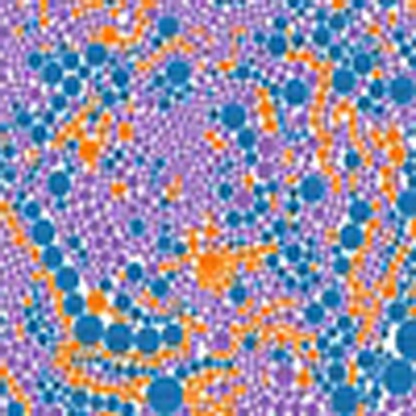

Omics repositories such as the NCBI Gene Expression Omnibus (GEO)^1^ and EBI ArrayExpress^2^ accumulate and serve gene expression data from thousands of studies. It is clear that these data contain much more information than what has typically been extracted from each individual dataset for the accompanying initial publication. However, currently, performing integrative analysis of large collections of gene expression studies to obtain a global integrated view of cellular regulation requires a significant data wrangling effort, that is, manually unifying data formats, adding metadata and converting the data to be more machine readable.

Due to high cost, gene expression profiling data are typically produced on a small scale, in targeted studies that are diverse with respect to tissue or cell type, genetic or chemical perturbation, disease model, expression assay platform and model organism. When submitted into public repositories such as GEO, the requirement for metadata annotation is minimal. Lack of standards for extensive metadata collection, and the diversity of individual studies, prohibits the easy reuse and integration of this type of data.

One of the advantages of carefully annotating studies from databases such as GEO is the potential for developing a signature search engine that operates at the data level. Tools such as SIGNATURE^3^, SPIED^4^, Cell Montage^5^, ProfileChaser^6^, ExpressionBlast^7^ and SEEK^8^ automatically attempt to compute differentially expressed signatures from GEO to provide a signature search engine at the data level. However, these tools are prone to mistakes because they automatically select the control and perturbation samples, as well as other aspects of signature generation and annotation, without relying on an extensive high-quality gold standard, which is needed for training better-quality classifiers.

Manual extraction of collections of gene expression signatures from GEO has been demonstrated to be highly useful. It was applied for drug repurposing^9^, suggesting novel drugs for many diseases^10^, and explaining mechanisms of action for many approved drugs^11^. Several efforts have attempted to further annotate datasets from GEO manually; one example is Gene Expression data Mining Toward Relevant Network Discovery (GEM-TREND)^12^. The disadvantage of manual curation is that it does not scale up to cover the thousands of studies currently available. For similar challenges, crowdsourcing projects have been developed as a potential solution to overcome this obstacle.

Crowdsourcing projects fall into two categories: microtasks and megatasks^13^,^14^. Microtasks consist of relatively trivial tasks that require a large number of participants; for example, extracting features from images of cells^15^. Crowdsourcing microtask projects in biomedical research have been established to improve automated mining of biomedical text for annotating diseases^16^, curation of gene-mutation relations^17^, identifying relationships between drugs and side-effects^18^, drugs and their indications^19^, as well as annotation of microRNA functions^20^. These efforts produce large collections of high-quality datasets that can be further utilized by algorithms that can extract new knowledge from already-published data that require better annotation, cleaning and reprocessing.

When computing gene expression signatures, the computational method used to identify the differentially expressed genes (DEGs) has a significant impact on the results. Using several benchmarks, including matching expression changes after transcription factor perturbations with ChIP-seq data, we previously showed that a method we developed called the Characteristic Direction (CD) significantly improves the prioritization of differentially expressed genes^21^ when compared with several commonly applied methods such as fold change, *T*-test or ANOVA, SAM^22^, *limma*^23^ or DESeq^24^.

In this study, we present the results of a crowdsourcing microtask project implemented to annotate and extract gene expression signatures from GEO. Our analysis of the crowdsourced gene expression signatures demonstrates that our collection of signatures is of high quality and can be used to recover prior knowledge, as well as discover new knowledge, about associations between drugs, genes and diseases. We also develop a web portal for users to visually identify associations between signatures, download the signatures for further computational analyses, and search the collections of gene expression signatures created for this project with their own signatures or by keywords. To scale up the collection of signatures for the three themes: disease, drug and gene perturbation, we use the manually extracted signature collections as a gold standard to train classifiers that automatically extract signatures from GEO.

## Results

### Crowdsourcing gene expression signatures

**Figure 1:**
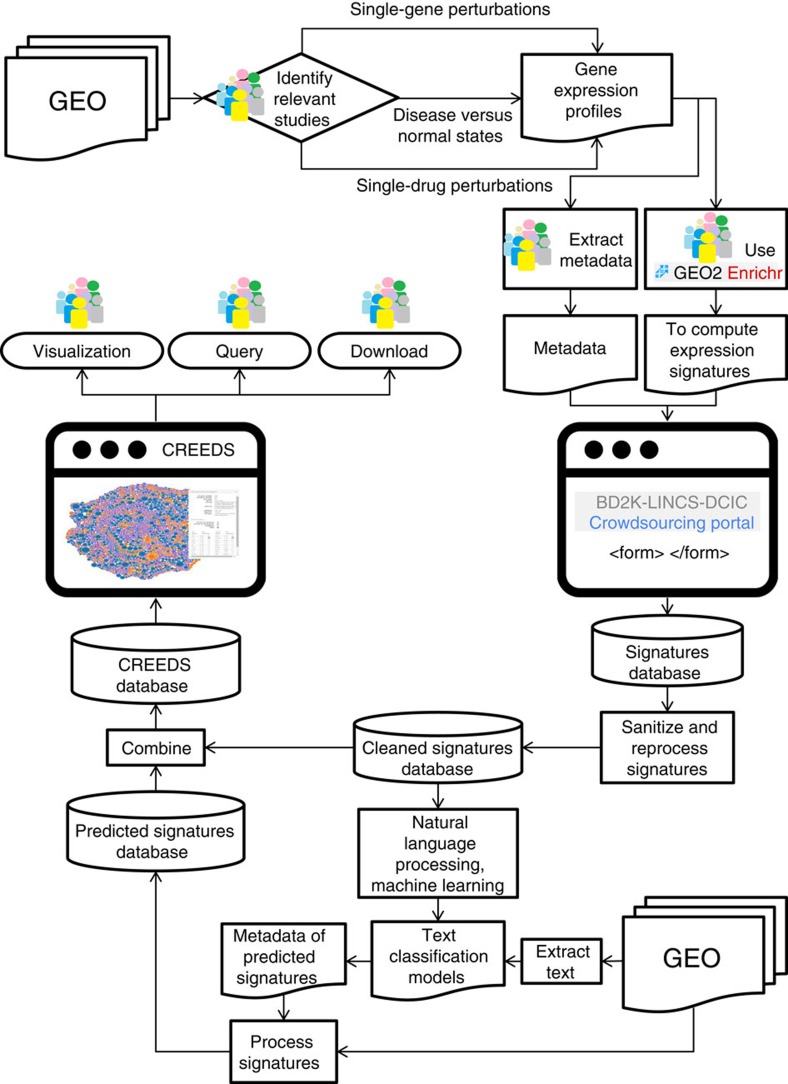
Workflow of the crowdsourcing project. Participants identify relevant studies from GEO and then extract gene expression signatures using GEO2Enrichr. Participants also add metadata to each signature. Submitted signatures were manually reviewed and then used to scale up the collections with machine learning methods. All signatures are served on the CRowd Extracted Expression of Differential Signatures (CREEDS) web portal.

The crowdsourcing challenge we designed followed several steps and consisted of several components and processes (Fig. 1). First, participants were asked to identify GEO studies in which single-gene or -drug perturbations were applied to mammalian cells, or in which normal versus diseased tissues were compared. After identifying relevant studies, participants extracted metadata from the studies and computed differential expression using GEO2Enrichr^25^, a Chrome extension we developed that makes the signature extraction process easy for non-experts. Extracted signatures were stored in a local database and sanitized by automated filters and manual inspection for improving accuracy and quality. The cleaned database of extracted signatures was used to visualize and analyse these signatures on the CRowd Extracted Expression of Differential Signatures (CREEDS) web portal. To scale up the collections, the human-extracted signatures were used as a gold standard for training machine learning classifiers for automated signature extraction. To date, the manual component of the signature database contains 3,100 submissions for single-gene perturbations, covering 1,186 genes from 1,635 studies; 1,081 disease signature submissions covering 450 diseases from 748 studies; as well as 1,238 submissions for drug perturbations covering 343 drugs from 443 studies (Supplementary Fig. 1a). After sanitizing the collections of signatures, a total of 2,177; 828 and 1,221 unique and valid signatures remained in the CREEDS database for single-gene perturbations, disease signatures, and drug perturbation signatures, respectively. The automated expansion of the signatures resulted in an additional set of 8,620 single-gene, 1,430 disease and 4,295 single-drug signatures extracted from 2,543 GEO studies.

We observe a skewed distribution with a long tail for the number of submissions per contributor (Supplementary Fig. 1b). A few enthusiastic curators contributed many more signatures than most others. The median number of signatures submitted per person was 16. We found no significant correlation between the number of signatures submitted per user and the quality of submissions (Supplementary Fig. 1c, Spearman's *ρ*=−0.08, *P* value =0.42). The leaderboard generally incentivized volunteers to submit more gene expression signatures. We found a significant negative correlation (Spearman's *ρ*=−0.64, *P* value<8.0e^−51^) between the scaled ranks of contributors and the number of newly submitted studies per day (Supplementary Fig. 1d). This suggests that highly ranked curators were inclined to continue to submit more.

### Quality improvement of crowdsourced gene expression signatures

To improve the quality of the gene expression signatures derived from thousands of GEO studies, we first checked for batch effects. To achieve this, we obtained the ‘scan date' from the raw microarray data files as an indicator of a potential source for batch effects. We then estimated the magnitude of such batch effect using principal variation component analysis^26^,^27^. We estimate that batch effects on average account for ∼18.7% of the variance in the gene expression dataset collections, whereas the perturbation versus control on average accounts for ∼16.7% of the variance (Supplementary Fig. 2a).

**Figure 2:**
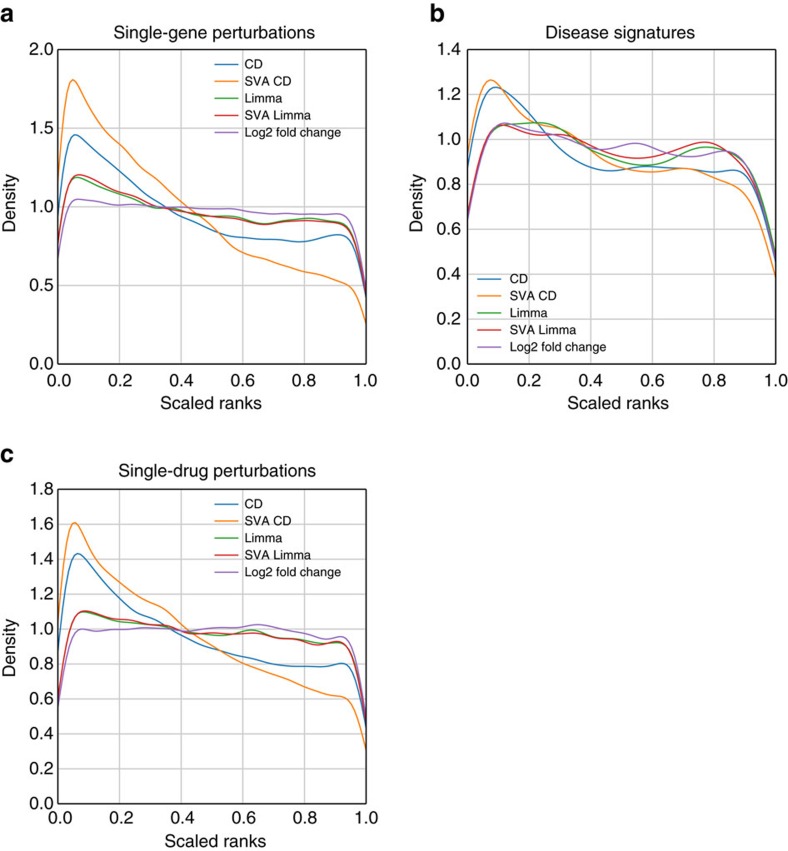
Batch effect correction influence on the quality of gene expression signatures. Line plots show the probability density distribution of the scaled ranks of expected DEGs in gene expression signatures from the three collections: (**a**) single-gene perturbations, (**b**) disease signatures, and (**c**) single-drug perturbations. The colours indicate which algorithm was used to call the differentially expressed genes: Characteristic Direction (CD), *limma*, or fold change; and whether batch effect correction was applied with surrogate variable analysis (SVA).

To correct for these batch effects, we applied the surrogate variable analysis (SVA)^28^ algorithm and generated new signatures using both the CD and *limma* methods to call the DEGs. To benchmark the quality of these signatures with or without the batch correction, we used collections of genes that are expected to be differentially expressed: direct protein interactions for gene perturbation, disease-gene associations for disease signatures, and targets of drugs for the drug-induced signatures. We observe that the batch correction improves the signal and quality of signatures (Fig. 2). We also found that the CD method outperformed *limma* in ranking the expected DEGs with these benchmarks.

### Comparing the collections with other similar resources

Next, we compared the collection of the crowdsourced gene expression signatures with MSigDB^29^, which contains 8 collections of gene sets. The collection C2 has curated gene sets extracted manually from tables and figures within publications. We compared the Chemical and Genetic Perturbations (CGP) subset within C2 from the latest version of MSigDB (v5.1) with our collections of signatures. The CGP subset has 3,396 gene sets, 33% of which have GEO identifiers (GSE) (Supplementary Fig. 3a). We first compared the overlapping GSEs and found that our collection covers 2,066 microarray studies, whereas the CGP subset covers 361 microarray studies with 54 shared studies (Supplementary Fig. 3b). Breaking down the overlap into the three collections, the shared GSEs with MSigDB are 31, 21 and 7 for the gene, disease and drug perturbations, respectively (Supplementary Fig. 3b). To compare the concordance of the gene-set for the 31 shared gene perturbations, we plotted the cumulative distribution from uniform distribution of the scaled ranks of the genes from our collection and those matching from MSigDB, and found that these gene sets are significantly similar (Supplementary Fig. 3c). Overall, we find that the MSigDB signatures overlap significantly with matched crowd-generated signatures, with only a few exceptions (Supplementary Fig. 3d, Supplementary Table 1). The discrepancies were due to a figure from He *et al.*^30^ that only reported genes related to the cell-cycle as opposed to all DEGs; the Sagiv *et al.*^31^ study reported DEGs in both siRNA knockdown and mAb treatment, whereas the DEGs in our database were derived from knockdown versus control only; and the gene sets curated from Soucek *et al.*^32^ by MSigDB do not match the original figure from that paper. However, overall, our analysis shows strong agreement between the matched signatures in both databases.

### Assessment of signature associations within each collection

**Figure 3:**
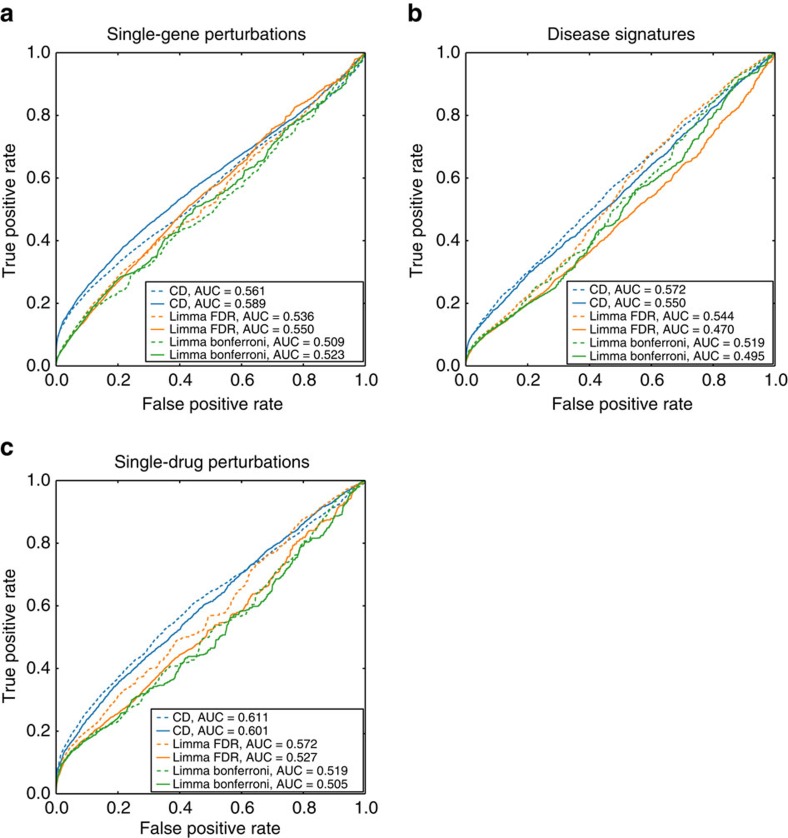
Benchmarking signature connections with prior knowledge. Signed Jaccard index and absolute Jaccard index are used to measure the similarity between signatures, and plotted in dashed and solid lines, respectively. Different methods for identifying differentially expressed genes include: the Characteristic Direction (CD), *limma* with Benjamini–Hochberg (BH) correction, and *limma* with Bonferroni correction. These are plotted in blue, orange and green, respectively. ROC curves are plotted for (**a**) recovering the same perturbed genes; (**b**) recovering similar diseases; and (**c**) recovering drugs with similar chemical structure.

We next asked whether signature similarity within and across the three collections can recover prior knowledge and discover novel connections. To globally assess associations between signatures within each collection, we used various methods to compute similarity between all pairs of signatures, and compared ranked signature associations with prior knowledge. Our results show that all of the three signature collections recover prior knowledge associations between genes, drugs and diseases (Supplementary Tables 2–4), and these associations are more discernable when computing differential expression with the CD method (Fig. 3). For example, individual independent studies that perturbed *Prkag3* by either knockout or gain-of-function mutation were identified as opposing signatures^33^ (Supplementary Table 2). An example that emerged from comparing disease signatures was the high similarity between hypercholesterolaemia and hepatocellular carcinoma signatures (Supplementary Table 3). It was shown that cholesterol metabolism is indeed deregulated in hypercholesterolaemia and hepatocellular carcinoma^34^,^35^. There are some top-ranked drug pairs that induce similar gene expression changes. For instance, the gene expression signatures for diethylstilbestrol, estradiol and tamoxifen from independent studies are very similar (Supplementary Table 4). The confirmation with prior knowledge associations suggests that we can predict novel associations with these data. In other words, top-ranked associations or top-ranked opposing signatures between drugs, diseases or genes that do not have literature support should be considered as high-quality predictions. Given the observation that drugs with highly similar chemical structure induce slightly more similar gene expression signatures than expected by chance (Fig. 3c), we further investigated whether the correlation between chemical similarity and gene expression signature similarity also applied to drugs pairs with lower chemical similarity scores. By binning the signed Jaccard index by Tanimoto coefficients, we found no correlation between lower chemical similarity and gene expression signature similarity (Supplementary Fig. 4), suggesting that partial chemical similarity is not predictive of expression similarity.

### Signature associations across the three collections

**Figure 4:**
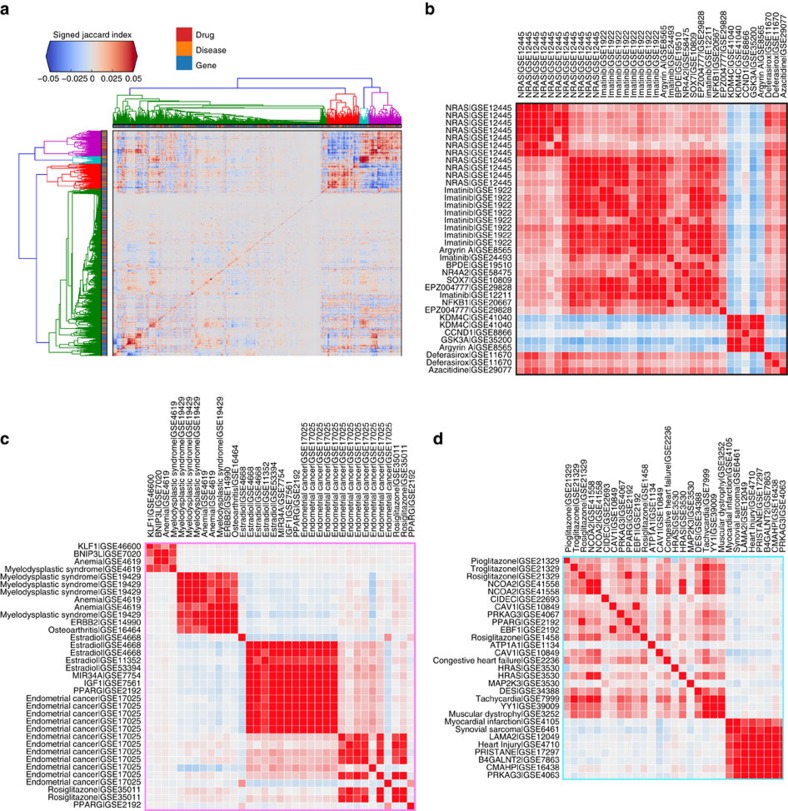
Hierarchical clustering of the adjacency matrix of all gene expression signatures and selected clusters. (**a**) The entire adjacency matrix of all signatures. (**b**–**d**) Three selected zoomed-in views of clusters from the adjacency matrix displayed in (**a**).

Using the signed Jaccard index, we computed an adjacency matrix for all possible pairs of signatures from the three collections (Fig. 4a) and observed many clusters. These clusters are heterogeneous, containing connections between genes, diseases and drugs. We highlight a few of these clusters (Fig. 4c,d), while others can be explored using the interactive clustergram or packed circles plot on the CREEDS web portal. In the first cluster that we chose to highlight, imatinib, a small molecule that is known to be a tyrosine kinase inhibitor^36^, has signatures that were generated from multiple cell lines, including K562 leukaemia cell line (GSE1922), chronic myelogenous leukaemia (CML) CD34+ cells (GSE12211) and three other CML cell lines (KU-812, KCL-22, JURL-MK1) (GSE24493), which cluster together with knockdown signatures of *NRAS* in melanoma cell lines (GSE12445) (Fig. 4b). This strongly suggests that *NRAS* is targeted by imatinib. Although *NRAS* is currently not considered a direct target of imatinib, a recent study showed that melanoma patients with *NRAS* mutations are resistant to imatinib therapy^37^. This raises the possibility that the wild-type form of *NRAS* is at least a key downstream effector of imatinib.

In the second cluster that we chose to highlight, multiple myelodysplastic syndrome (MDS) signatures from CD34+ cells (GSE4619, GSE19429) and *ERBB2* overexpression signature from MCF10A cells (GSE14990) cluster together (Fig. 4c), suggesting that the up-regulation of *ERBB2* may have a role in MDS. Indeed, it was shown that *ERBB2* amplification is present in 35% of a cohort of MDS patients^38^. In the third example, endometrial cancer signatures (GSE17025) are shown to cluster with estradiol signatures derived from MCF7 cells from multiple independent studies (GSE4668, GSE11352, GSE53394), as well as *MIR34A* overexpression signature from HCT116 cells (GSE7754), *PPARG* overexpression signature from NIH-3T3 cells (GSE2192), and IGF1 stimulation signature from MCF7 cells (GSE7561) (Fig. 4d). Estradiol has been shown to increase the risk for endometrial cancer^39^,^40^ and was previously discovered in a meta-analysis study of this disease^41^. Insulin-like growth factor 1 (*IGF1*) and its receptor *IGF1R* are known to be indirectly activated by estradiol^42^,^43^,^44^. Downstream of the *IGF1R* receptor phosphoinositide kinase 3 (*PI3K*), the mammalian target of rapamycin (*mTOR*) and MAPK signalling promote protein synthesis, cell growth, and cell proliferation, potentially driving the progression of endometrial cancer^45^,^46^. Peroxisome proliferator-activated receptor gamma (*PPARG*) has also been shown to induce the development of multiple types of cancers^47^, and it is known to play a role downstream of adiponectin during insulin resistance^48^, which is a significant risk factor for endometrial cancer^49^. The fourth cluster contains a *YY1* knockout (GSE39009) signature produced in mice soleus, and an autosomal muscular dystrophy signature from a mouse model sourced from the diaphragm (GSE3252). This association suggests that *YY1* may be disrupted in muscular dystrophy tissues. Literature supports that almost all facioscapulohumeral muscular dystrophy patients carry deletions of repetitive elements (D4Z4) that contain binding sites for *YY1*^50^,^51^. All of the aforementioned examples are just a small portion of the signature connections our integrative analysis offers. These examples illustrate how novel associations between diseases, genes and drugs can be discovered through a crowdsourcing project.

### Identifying drug mimickers

**Figure 5:**
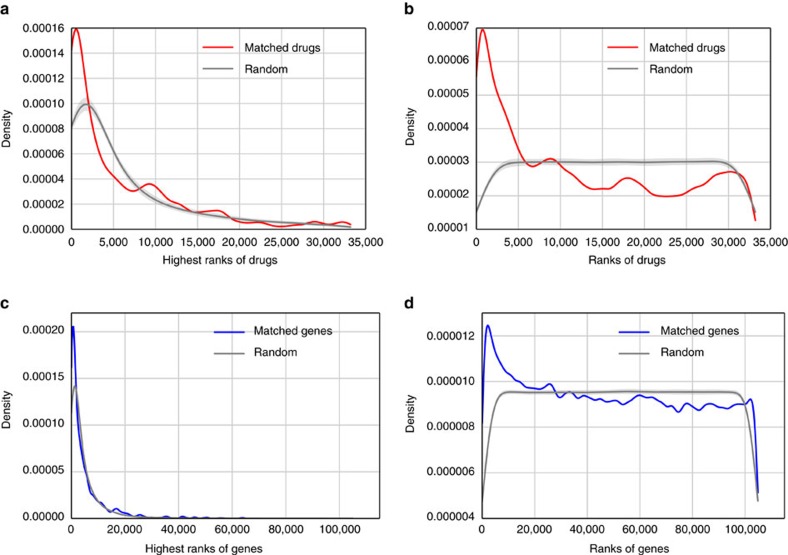
Distributions of the ranks of matched perturbations between signatures from CREEDS and the LINCS L1000 dataset. The highest ranks (**a**,**c**), and all ranks (**b**,**d**) of matched drugs (**a**,**b**) and matched genes (**c**,**d**) are presented. Drug perturbation signatures from CREEDS were queried against ∼30,000 significant drug perturbation signatures from the LINCS L1000 dataset; whereas gene perturbation signatures from CREEDS were queried against ∼110,000 gene perturbation signatures from the LINCS L1000 dataset.

To further demonstrate the utility of the crowdsourced gene expression signatures of drug perturbations, we queried these signatures against the database of drug or other small molecule compound signatures derived from the LINCS L1000 dataset. We then recorded the ranks of the matched drugs out of >30,000 LINCS L1000 signatures and found that many crowdsourced drug perturbation signatures are significantly highly ranked (Rank sum *P* value <4.8e^−24^) (Fig. 5a,b, Table 1). Similarly, the results can also be reproduced when querying the drug perturbation signatures against >6,000 signatures from the Connectivity Map dataset^52^ (Supplementary Fig. 5). We additionally queried the gene perturbation signatures against 109,000 shRNA knockdown and over-expression profiles from the LINCS L1000 data and found similar consistency (Fig. 5c,d). These results suggest that some drugs induce similar transcriptional changes in small-scale studies, when compared with results from large-scale studies such as LINCS L1000 and the original Connectivity Map. This means that we can identify potential mimickers using the LINCS L1000 dataset for drugs whose signatures are highly similar between the LINCS L1000 dataset and the GEO studies. Interestingly, we found that dexamethasone signatures in the LINCS L1000 dataset were ranked in the top 10 using dexamethasone-induced gene expression signatures from three independent GEO studies: GSE34313, GSE7683 and GSE54608 (Supplementary Table 5). The three studies treated dexamethasone in different cell types: human airway smooth muscle cells, mice primary chondrocytes, and in a human oviductal cell line, suggesting that the effect of this glucocorticoid agonist is robust across mammalian cells. Among the top-ranked potential mimickers of dexamethasone, flumetasone and betamethasome are both corticosteroids indicated for inflammation, confirming that the approach is able to identify drugs with similar physiological effects. Moreover, we found a small molecule compound 5,6-epoxycholesterol (BRD-K61480498) with gene expression profiles highly similar to that of dexamethasone. 5,6-epoxycholesterol also has a similar chemical structure, but unknown anti-inflammatory effects. As such, it is an example of a strong candidate for further experimental validation.

### Web portal to visualize and query the signatures database

To provide easier access to the three collections of the gene expression signatures for knowledge reuse and exploration, we developed a web portal (Supplementary Fig. 6). This portal visualizes all of the signatures in a packed circles layout in which similar signatures are closer to each other. Furthermore, the portal has interactive heatmaps of hierarchically clustered matrices of all signatures. The web portal is available at: http://amp.pharm.mssm.edu/creeds. The portal also has a search engine that enables users to search by text or by providing lists of up and down DEGs. Since DEGs for the gene expression profiles in the CREEDS database were computed with the CD method, which is not a standard method, we tested whether signatures computed via other methods would produce similar results. We found that most signatures computed by fold change or *limma* are ranked similarly (Supplementary Fig. 7). However, some signatures were not ranked as expected. The CD is a multivariate method, whereas fold change and *limma* are univariate; a gene can be identified as significantly differentially expressed by a univariate method but may not contribute to the joint expression changes of large sets of genes.

Finally, to scale up the three collections of signatures, we developed machine learning classifiers that use the manually curated signatures as a training set. The classification task was divided into two parts: (1) classify whether a GEO dataset is likely to contain gene, disease or drug signatures, and (2) label the samples as control and perturbation. The features for the classifiers were extracted from the text associated with the each GEO study in our manually curated collection as well as from all currently available studies on GEO where genome-wide expression was assessed by microarrays to profile human, mouse or rat cells and tissues. Overall, we observe that various classifiers perform very well (Supplementary Fig. 8).

We next asked whether we have collected a sufficient number of manually curated studies or whether more manual curation could improve the performance of the classifiers. We see, for example, that Naïve Bayesian classifiers no longer improve once ∼1,000 annotated studies are used for each collection category (Supplementary Figs 9–13). With these machine learning classifiers, we automatically identified a large collection of additional signatures for the three collections. In total, this process enabled us to add 8,620 gene; 4,295 drug and 1,430 disease automatically extracted signatures. Each signature carries a *P*-value for confidence, and all these signatures are available for download and search on the CREEDS web portal.

## Discussion

Gene expression profiling is arguably the most common type of omic data. The resource we developed for this project can be combined with transcriptomics profiling projects such as Genotype-Tissue Expression^53^, the Cancer Genome Atlas^54^, the Cancer Cell-Line Encyclopaedia^55^, and the Library of Integrated Network-based Cellular Signatures (LINCS). Here we show, for example, how combining drug perturbation signatures collected from GEO with the LINCS L1000 data can be used to identify potential novel drug mimickers.

The manually extracted and cleaned signatures were proven to be useful as a training set that enabled us to scale up the three collections of signatures using machine learning. However, we are aware that the quality of the automatically generated signatures is not as good as the signatures created by the human annotators. One solution to improve the process is to intelligently integrate machine learning with crowdsourcing by using active learning. With active learning, unlabelled instances are presented to human annotators with suggestions; this allows the classifiers to be improved dynamically while reducing the effort required of the curators^56^. Active learning methods have been shown to achieve improved performance in similar settings^57^,^58^.

This project highlights the commitment of citizen scientists to spare their time in pursuit of a common goal that can advance science and medicine. Indeed, we show how this collective effort was used to identify novel relationships between genes, drugs and diseases. While we highlighted several top predictions that emerged from our analysis, many more hypotheses can be formed by interacting with the CREEDS portal at: http://amp.pharm.mssm.edu/creeds.

## Methods

### Extracting gene expression signatures from GEO by the crowd

Three crowdsourcing microtasks were established to collect gene expression signatures from GEO. These are: single-gene perturbations, comparison between diseased and normal tissues, and single-drug perturbations. These three types of signatures were extracted using the Google Chrome extension GEO2Enrichr^25^ and submitted through the BD2K-LINCS-DCIC Crowdsourcing Portal at: http://www.maayanlab.net/crowdsourcing/. These crowdsourcing tasks were open to all participants, but a significant majority of the contributors were students from the massive open online course Network Analysis in Systems Biology 2015 (NASB2015) offered on the Coursera platform. These participants were given detailed instructions for finding, labelling, and extracting gene expression profiles from GEO. Participation was strictly voluntary, and was not required for completion of any parts of the course. Participants were not provided with a list of predefined gene expression profiles; instead, they were encouraged to find diverse, yet relevant, gene expression studies from GEO. Briefly, contributors first had to locate relevant GEO studies fitting into one of the three themes, and then select the perturbation and control samples (GSMs) from GEO series (GSE) or GEO datasets (GDS). Only gene expression studies from selected species of mammals (human, mouse and rat) were considered valid. Participants were also asked to submit additional metadata about the cell or tissue type, and gene, disease or drug used in each experiment and associate these with common published identifiers. Standard names of genes, diseases, and drugs were provided as autocomplete options in the submission forms, created from controlled vocabularies: HGNC for genes^59^, disease names from the Disease Ontology^60^ and drug names from DrugBank^61^. To incentivize participants, a real-time leaderboard was developed to display the number of submissions from each user, and modest prizes were promised to the top ten contributors (custom T-shirt and headphones). Additionally, co-authorship on the published research resulting from these crowdsourcing tasks was promised to contributors of a minimum of 15 valid entries.

### Sanitization of the crowdsourced gene expression signatures

Multiple steps of quality control filters were applied to improve the collection of the gene expression signatures extracted by the crowd. We first performed integrity checks using the association between GEO studies (GSE or GDS) and samples within these studies (GSMs) by re-processing all the collected gene expression signatures based on the metadata supplied by the curators. Signatures in which GSMs did not match their GSE or GDS, as well as signatures with the same GSMs in the control and perturbation groups, were automatically detected and removed. The next filter was applied only to the single-gene perturbation collection. We checked whether gene symbols submitted by the curators are valid HGNC gene symbols, removing all entries with invalid genes. The next filter was semi-automatic: we corrected signatures in which the control and perturbation samples were switched. Our final filter was to manually check if the submitted signatures agree with the descriptions associated with the original GEO studies. After applying each of these filters, we recorded the number of invalid submissions by curators and removed the submissions from any curators who had submitted more than 10% invalid signatures. As a result, ∼20% of all the submissions were removed from the final collections.

### Evaluation of batch effects

To obtain batch information from each study, we retrieved the ‘scan date' from the raw microarray CEL files and assumed that the experiments were performed on the same dates that were listed within the experimental batch. We then quantified the batch effect using principal variation component analysis^26^,^27^, which attributes the variation in the gene expression data to known sources such as batches and experimental conditions. Batch effects were corrected using the surrogate variable analysis (SVA) algorithm^28^ implemented in R^62^ with default parameters.

### Construction of expected DEGs from prior knowledge

To generate lists of expected DEGs for the three collections of signatures for benchmarking, we used: (1) the known direct physical interactors of the protein product of a gene from a consolidated protein–protein interaction network we assembled for a previous study^63^; (2) a consolidated collection of manually-curated disease-gene associations from the DISEASES resource^64^; and (3) known drug targets from DrugBank v4.3^61^.

### Measuring similarity between signatures

To compare signatures, we abstracted signatures to sets of up- and down-regulated genes. The signed Jaccard index for two signatures *S*_*i*_ and *S*_*j*_ is defined as:


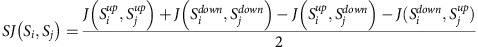


where *S*^*up*^and *S*^*down*^ denote the up- and down-regulated gene sets, respectively. The signed Jaccard index considers the direction when comparing a pair of gene expression signatures. It has a range of 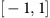 where 1 represents identical signatures, and −1 represents signatures of reverse effect, whereas 0 represents unrelated signatures.

Signature pairs from different GEO studies were ranked based on the signed Jaccard index. Prior knowledge from various resources about known connections between genes, diseases and drugs was used to examine whether signature similarity can be used to recover known associations between genes, drugs and diseases. Specifically, pairs of diseases were connected through the Disease Ontology^60^, and pairs of drugs were connected by the drugs' molecular structure fingerprints and considered similar if the Tanimoto coefficient was >0.9. Structural fingerprints were computed with the extended-connectivity fingerprints ECFP4^65^. To score the predictions of associations between genes, drugs and diseases, receiver operating characteristic (ROC) curves were plotted and the area under the ROC curve (AUC) was calculated. DeLong's test^66^ was performed to compare the difference between ROC curves.

### Natural language processing of text from GEO series

The text from each GEO series including title, summary, and keywords were extracted and processed separately. Text was first tokenized into words that were then lemmatized using the WordNet Lemmatizer^67^ and stemmed using the Porter stemming algorithm^68^. Term frequency-inverse document frequency (TF-IDF)^69^ was used to convert stems of both unigrams and bigrams into numerical values that measure the importance of an n-gram to a document in the context of the collection of documents. Truncated singular value decomposition was used to reduce dimensionality of the TF-IDF matrices to capture at least 10% of the variance. To visualize the GEO studies in the textural feature space, t-Distributed Stochastic Neighbour Embedding^70^ was used to reduce the dimensionality of the matrices from the truncated singular value decomposition. To classify whether a GEO series contains a disease signature, three textural feature matrices representing the title, summary and keywords were used to train and test a classifier. To measure the performance of the classification, three-fold cross-validation was applied to calculate the area under the ROC curve, area under the precision-recall curve, Matthew's correlation coefficient and F1 score. Classifiers from the scikit-learn^71^ package were tested including: random forest^72^, extra trees^73^, support vector classifier and the XGBoost implementation of gradient boosting machines^74^. Hyperparameters of the classifiers were optimized using grid search.

### Classifying control versus treatment samples based on text

We formulate the problem of classifying GEO samples as a binary classification problem. This means that we aim to learn from text-derived features whether a sample is part of the control or treatment group. Features were extracted from the following text fields associated with each GEO sample: title, description, characteristics and source name. These text elements were tokenized and converted to binary vectors representing the presence or absence of tokens for each sample. The classifier we used for solving this problem is a Bagging^75^ of 20 multinomial Bernoulli Naïve Bayesian^69^ classifiers after probability calibration with isotonic regression^76^. To measure the performance of the classifier, 10-fold cross-validation was applied to calculate area under the ROC curve, area under the precision-recall curve, Matthew's correlation coefficient and F1 score.

### Development of the CREEDS web portal

A web portal was developed for visualizing and querying the collections of the gene expression signatures. Relationships between all signatures are visualized using the D3.js pack layout and D3.js clustergrammer. Clustergrammer is a visualization tool we developed starting with the open-source code example for the matrix co-occurrence visualization on the D3.js website. All data and metadata of the signatures are stored in a MongoDB database. The portal uses the Python Flask framework. Signed Jaccard index was implemented to query signatures in which users input up or down gene lists into two separate text boxes. The text signature search option queries the metadata text of all signatures in the database. RESTful application programming interface (API) endpoints were also developed to enable users to programmatically query and search the CREEDS database.

### Automatic extraction of gene expression signatures from GEO

To automatically extract gene expression signatures from GEO, we first applied the gradient boosting machines classifier (described above) to predict the categories of all GEO studies (*n*=31,905) performed in human, mouse or rat using microarrays. The classifier utilized the title, summary and keywords from each study. After this step, we selected the studies that were predicted to be gene, disease or drug perturbations with a probability threshold greater than *P*>0.9. We then applied the Naive Bayesian-based classifiers described above to predict the probability of whether samples associated with these studies have controls based on the sample titles. Next, we computed the pairwise Manhattan distance between the samples based on features extracted from sample descriptive terms, and then used the DBSCAN^77^ algorithm with minimum samples set of 2 to perform clustering on the distance matrix between samples to identify clusters of semantically similar samples. We removed any clusters with large standard deviation (*P*>0.2) to reduce instances of mixture between control and perturbation samples. To determine whether a cluster of samples is a control group or a perturbation group, we chose the average probability *P*>0.7 and *P*<0.3 from the Naive Bayesian-based classifier as control group and treatment group, respectively. Next, we enumerated every pair of valid control groups and perturbation groups within each study as metadata for valid predicted gene expression signatures.

To properly label the terms associated with each predicted signature, we used the API of BeCAS^78^ to tag biological entities from the text associated with each study, as well as the text associated with the samples, including: genes, cell or tissue, disease, and drug or other small molecule chemical; and then recorded these term counts for a final decision of which terms we should use to label each signature. To process the gene expression data of the predicted gene expression signatures, we first used SVA^28^ to correct the batch effect as described above, and then applied the CD algorithm^21^ to compute differential expression.

### Data availability

All extracted and processed signatures with their accession numbers and other metadata are freely available for download from the CREEDS portal at: http://amp.pharm.mssm.edu/creeds. The CREEDS portal also provides the data through API. Users can search the data by submitting their own signatures for analysis. The site also provides two modes of visualization of all signatures. Accession codes for top hits for drug signatures extracted from GEO queried against drug perturbations can be found in Table 1.

## Additional information

**How to cite this article:** Wang, Z. *et al.* Extraction and analysis of signatures from the Gene Expression Omnibus by the crowd. *Nat. Commun.* 7:12846 doi: 10.1038/ncomms12846 (2016).

## Supplementary Material

Supplementary Information Supplementary Figures 1-13, Supplementary Tables 1-5

## Figures and Tables

**Table 1:** Top hits for drug signatures extracted from GEO queried against drug perturbations from the LINCS L1000 dataset processed using the Characteristic Direction method.

**Drug name**	**PubChem ID**	**GEO Accession**	**organism**	**GEO platform**	**Rank**
Dexamethasone	5743	GSE34313	human	GPL6480	1
Doxorubicin	31703	GSE58074	human	GPL10558	1
Azacitidine	9444	GSE29077	human	GPL571	1
Azacitidine	9444	GSE29077	human	GPL571	1
Azacitidine	9444	GSE29077	human	GPL571	1
Lapatinib	208908	GSE38376	human	GPL6947	2
Methylprednisolone	6741	GSE490	rat	GPL85	2
Lapatinib	208908	GSE38376	human	GPL6947	2
Dexamethasone	5743	GSE54608	human	GPL10558	3
Lapatinib	208908	GSE38376	human	GPL6947	3
Tretinoin	444795	GSE1588	mouse	GPL81	3
Methylprednisolone	6741	GSE490	rat	GPL85	3
Tretinoin	444795	GSE32161	human	GPL570	3
Methylprednisolone	6741	GSE490	rat	GPL85	3
Methylprednisolone	6741	GSE490	rat	GPL85	4
Trichostatin A	444732	GSE1437	mouse	GPL81	4
Dexamethasone	5743	GSE7683	mouse	GPL1261	5
Cycloheximide	6197	GSE8597	human	GPL570	5
Methylprednisolone	6741	GSE490	rat	GPL85	6
Sorafenib	216239	GSE39192	human	GPL6947	7
Vemurafenib	42611257	GSE37441	human	GPL10558	8
Methylprednisolone	6741	GSE490	rat	GPL85	10
Curcumin	969516	GSE10896	human	GPL570	14
Curcumin	969516	GSE10896	human	GPL570	15
Vemurafenib	42611257	GSE37441	human	GPL10558	15
Lapatinib	208908	GSE38376	human	GPL6947	16
Methylprednisolone	6741	GSE490	rat	GPL85	17
Tretinoin	444795	GSE1588	mouse	GPL81	20
Vemurafenib	42611257	GSE42872	human	GPL6244	23
Azacitidine	9444	GSE29077	human	GPL571	24
Troglitazone	5591	GSE21329	rat	GPL341	31
Decitabine	451668	GSE29077	human	GPL571	36
Vemurafenib	42611257	GSE37441	human	GPL10558	36
Thapsigargin	446378	GSE19519	human	GPL570	37
Methylprednisolone	6741	GSE490	rat	GPL85	48
