# Supplementary material for: Extraction and analysis of signatures from the Gene Expression Omnibus by the crowd
Source: Nat Commun. 2016 Sep 26;7:12846. doi: 10.1038/ncomms12846 (PMC5052684; doi:10.1038/ncomms12846)
Supplement: Supplementary Information — Supplementary Figures 1-13, Supplementary Tables 1-5 [file ncomms12846-s1.pdf]

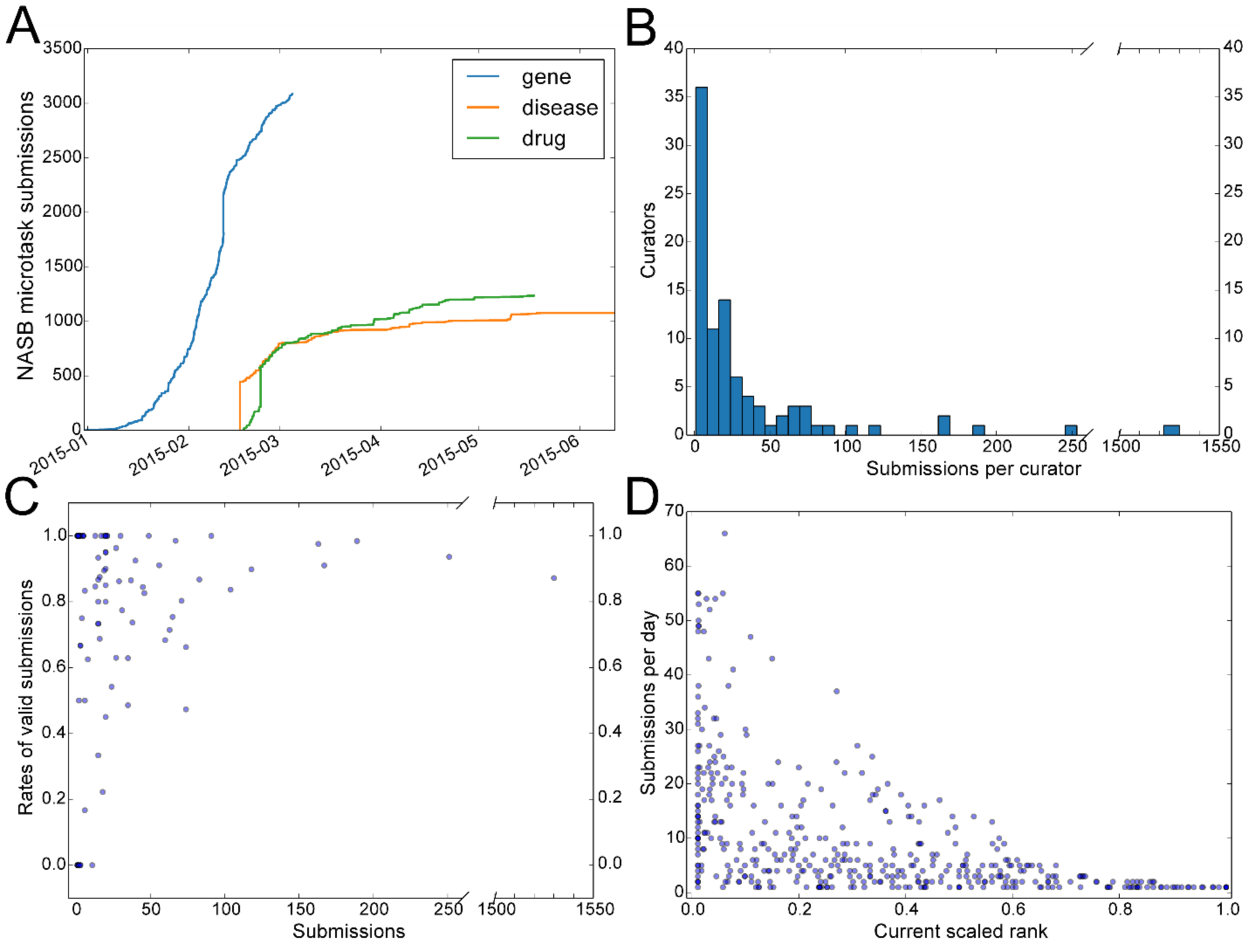

Supplementary figure 1. Descriptive statistics of the crowdsourced submissions. (A) Line chart showing the number of submissions over time; single-gene perturbations, disease signatures, and single-drug perturbations are plotted in blue, orange, and green, respectively. (B) Histogram of the distribution of signature submissions per curator. (C) Scatter plot of the relationship between the number of submissions per user and the number of valid submissions. (D) Scatter plot of the relationship between the leadership board ranks and the number of daily submissions made by each curator.

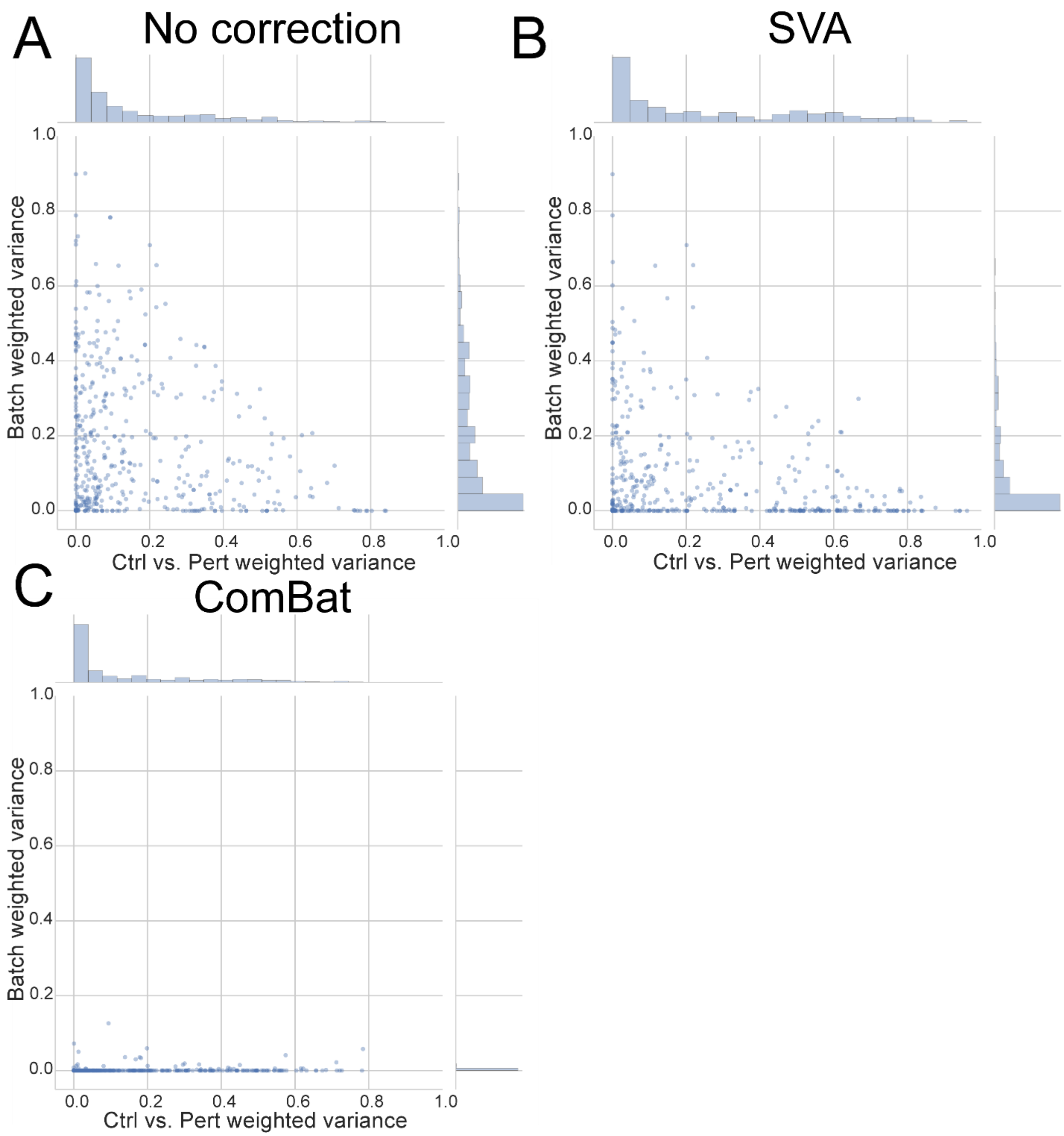

Supplementary figure 2. Quantification of batch effects. Scatter plots of the weighted variance attributed to batch effects vs. that attributed to the effects of the experimental perturbations. (A) Without correction; (B) Corrected by SVA; and (C) Corrected by ComBat. The histograms on top and right side of the scatter plots show the distribution of weighted variances.

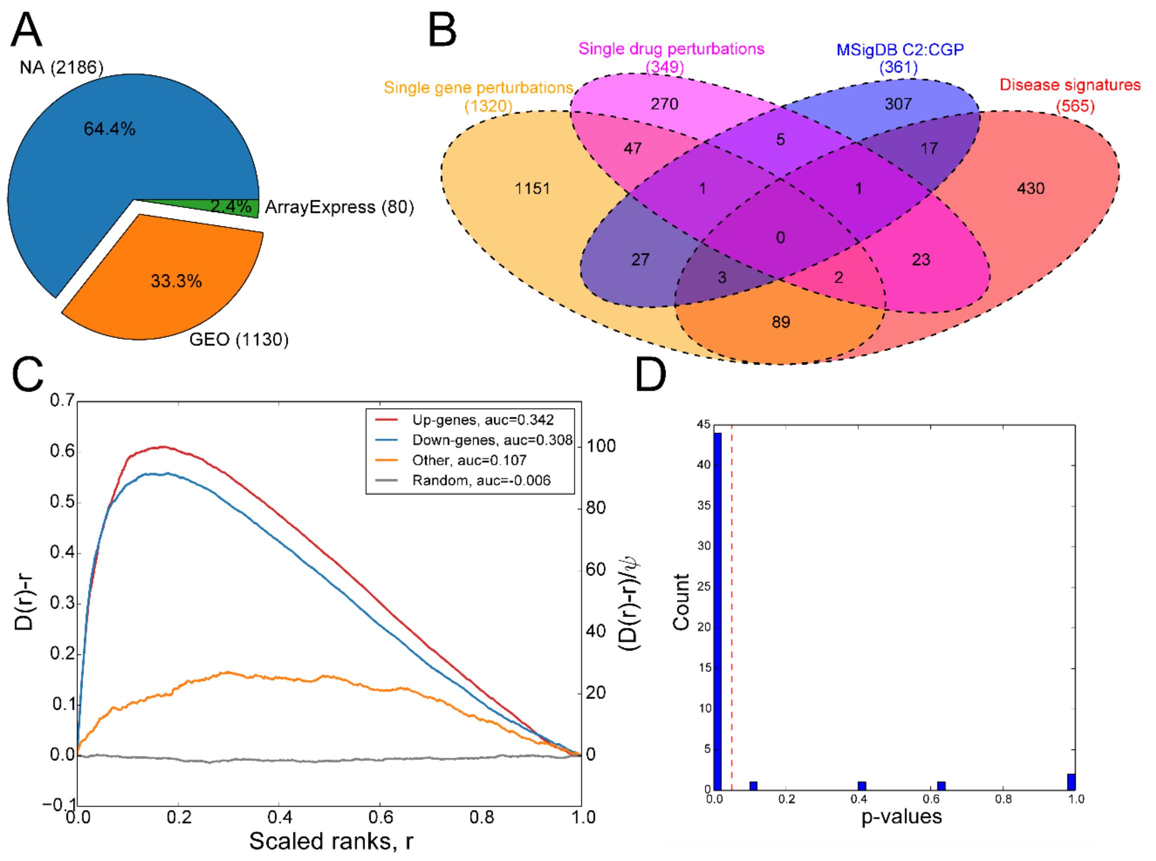

Supplementary figure 3. Comparison between curated signatures from MSigDB and CREEDS. (A) Pie chart showing the number of MSigDB curated gene sets from literature with the portion of corresponding publicly available microarray data. (B) Venn diagram showing the overlap of GEO studies among the three CREEDS collections, and the curated signatures in MSigDB. (C) Line chart showing the deviation of the cumulative distribution from uniform distribution for the scaled ranks of gene sets curated by MSigDB and their matched CREEDS signatures. (D) Histogram of Fisher's exact test p-values showing the number of matched signatures with significant overlaps between MSigDB and CREEDS.

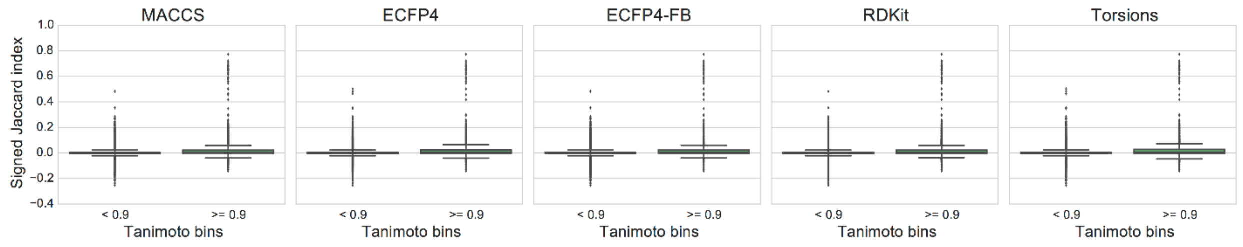

Supplementary figure 4. Chemical structural similarity and gene expression signature similarity. Boxplots show the distribution of signed Jaccard indexes of drug pairs that are highly and lowly structurally similar based on Tanimoto scores computed by different chemical structural fingerprint algorithms: MACCS, ECFP4, feature-based ECFP4, RDKit, and Torsions.

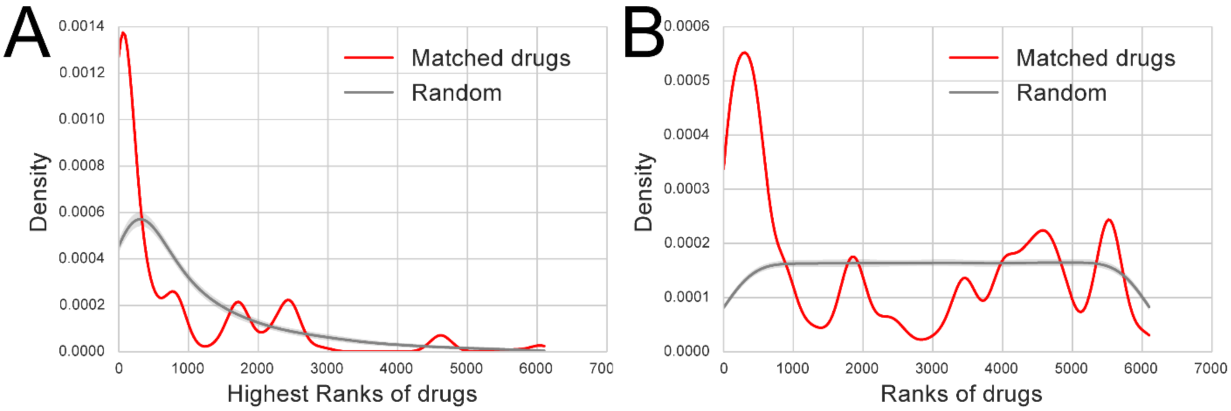

Supplementary figure 5. Distributions of the ranks of matched drug perturbations between signatures from CREEDS queried against signatures from the original initial version of the Connectivity Map (CMAP) dataset. The highest ranks and all of the ranks of the matched drugs when using drug perturbation signatures from CREEDS to query against the 6,100 drug perturbation signatures in the CMAP dataset are plotted in (A) and (B), respectively.

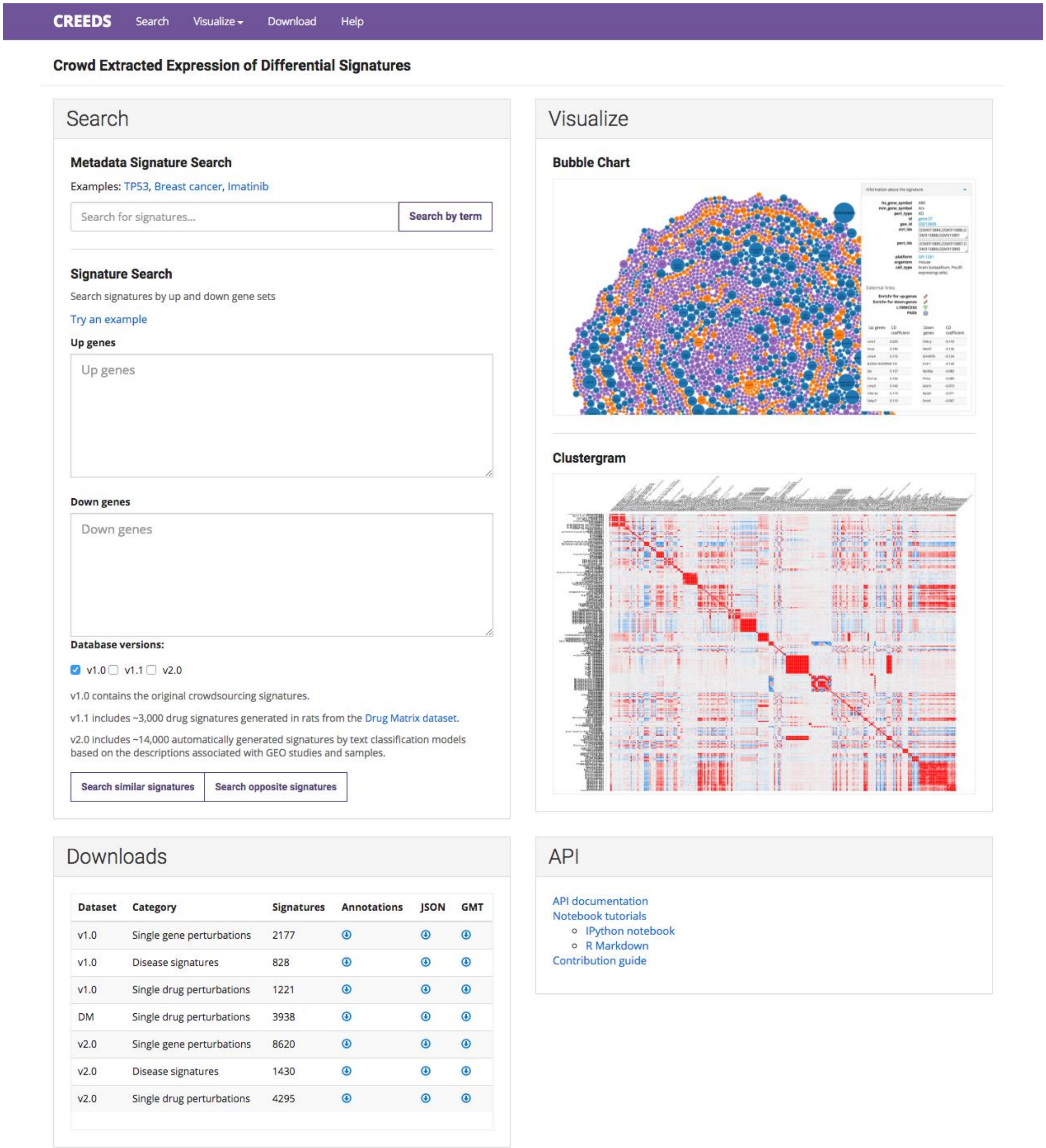

Supplementary figure 6. Screenshot from the landing page of the CREEDS portal (http://amp.pharm.mssm.edu/creeds).

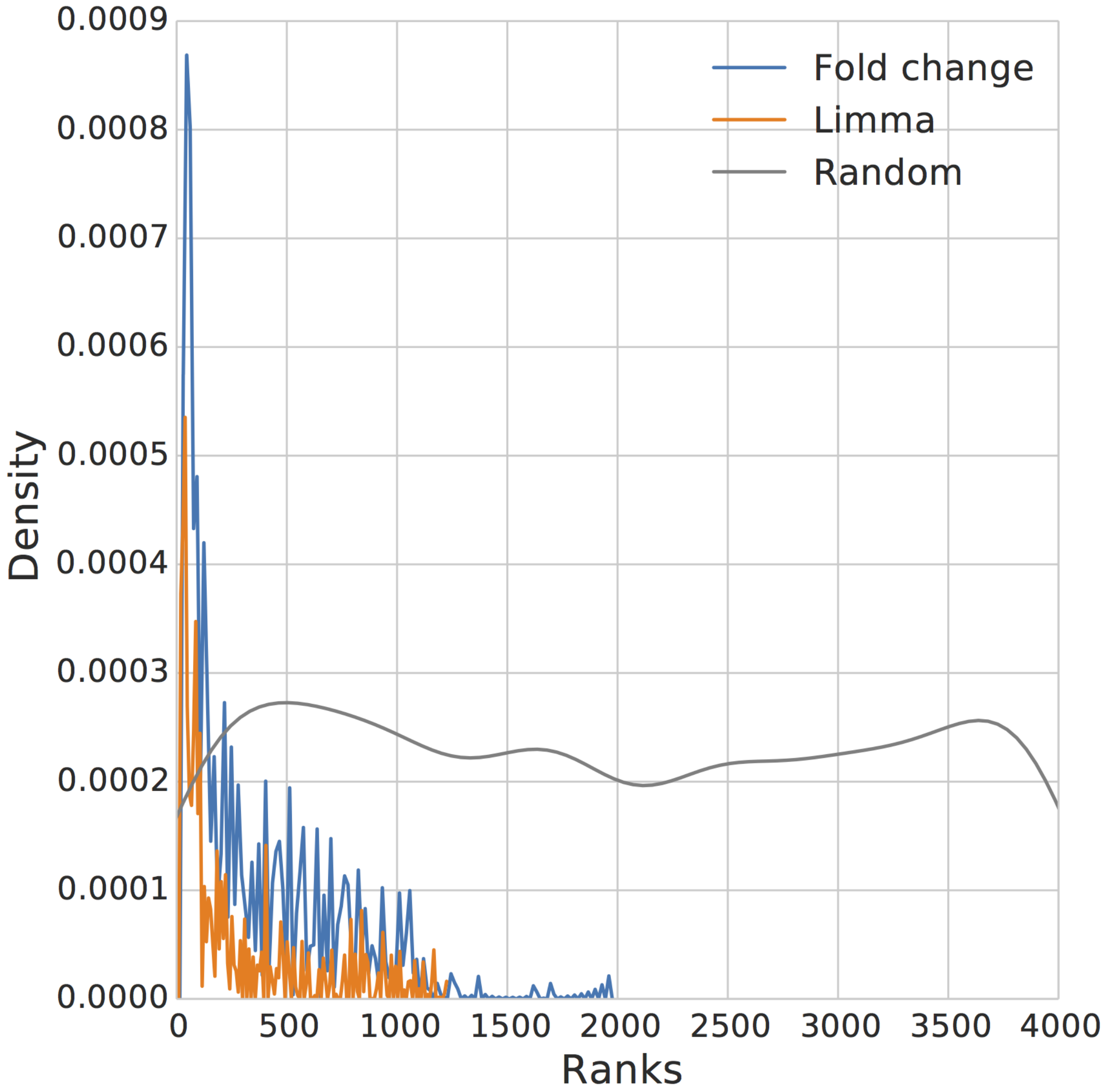

Supplementary figure 7. Comparing differential expression methods to query results against the CREEDS collection. The plot shows the distribution of ranks of matched signatures using the up or down genes identified by two different methods: fold change (blue) and Limma (orange) to create input of gene lists to be queried against the CREEDS database.

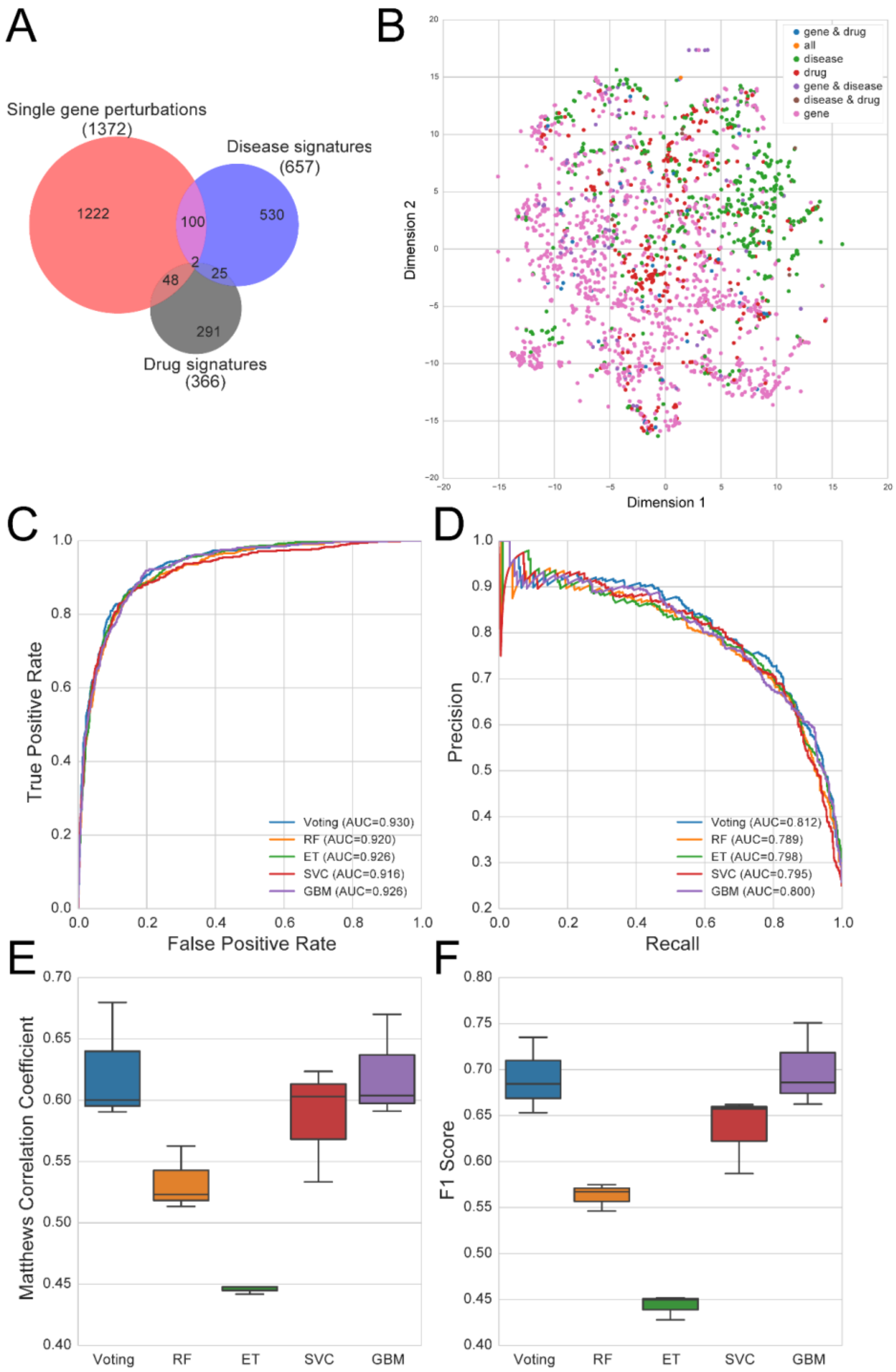

Supplementary figure 8. Natural language analysis of GEO studies using the extracted signatures as a training set. (A) Venn diagram showing the overlap between GEO series collected by the crowd for the three collections and used as a training set. (B) t-SNE visualization of textural features extracted from the summary of the GEO studies. The performance of different classifiers to categorize GEO studies as containing (or not) disease signatures (C-F). (C-F) Different evaluation metrics including AUROC, Area under the Precision-Recall Curve (AUPRC) Matthew's correlation coefficient, and F1 score. Abbreviations: Random Forest (RF), Extra Trees (ET), Support Vector Classifier (SVC), and Gradient Boosting Machine (GBM).

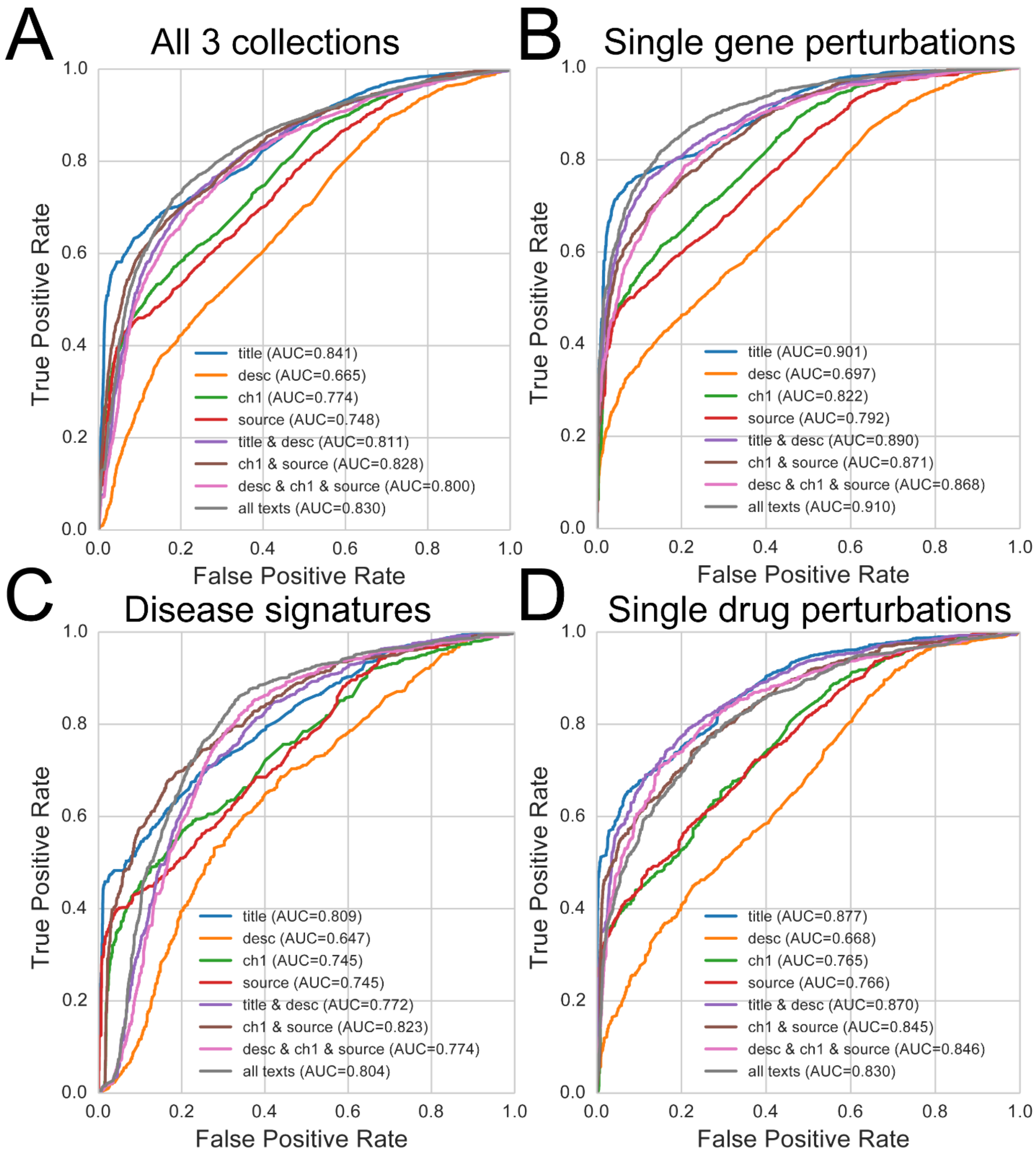

Supplementary figure 9. Performance evaluations of binary classifiers for predicting control vs. perturbation of samples. The ROC curves of different subsets of text features extracted from the "Title", "Description" (desc), "Sample characteristics" (ch1), and "Sample source name" (source) of samples (GSMs) from the GEO studies, as well as different combinations of those subsets, are plotted in different colors.

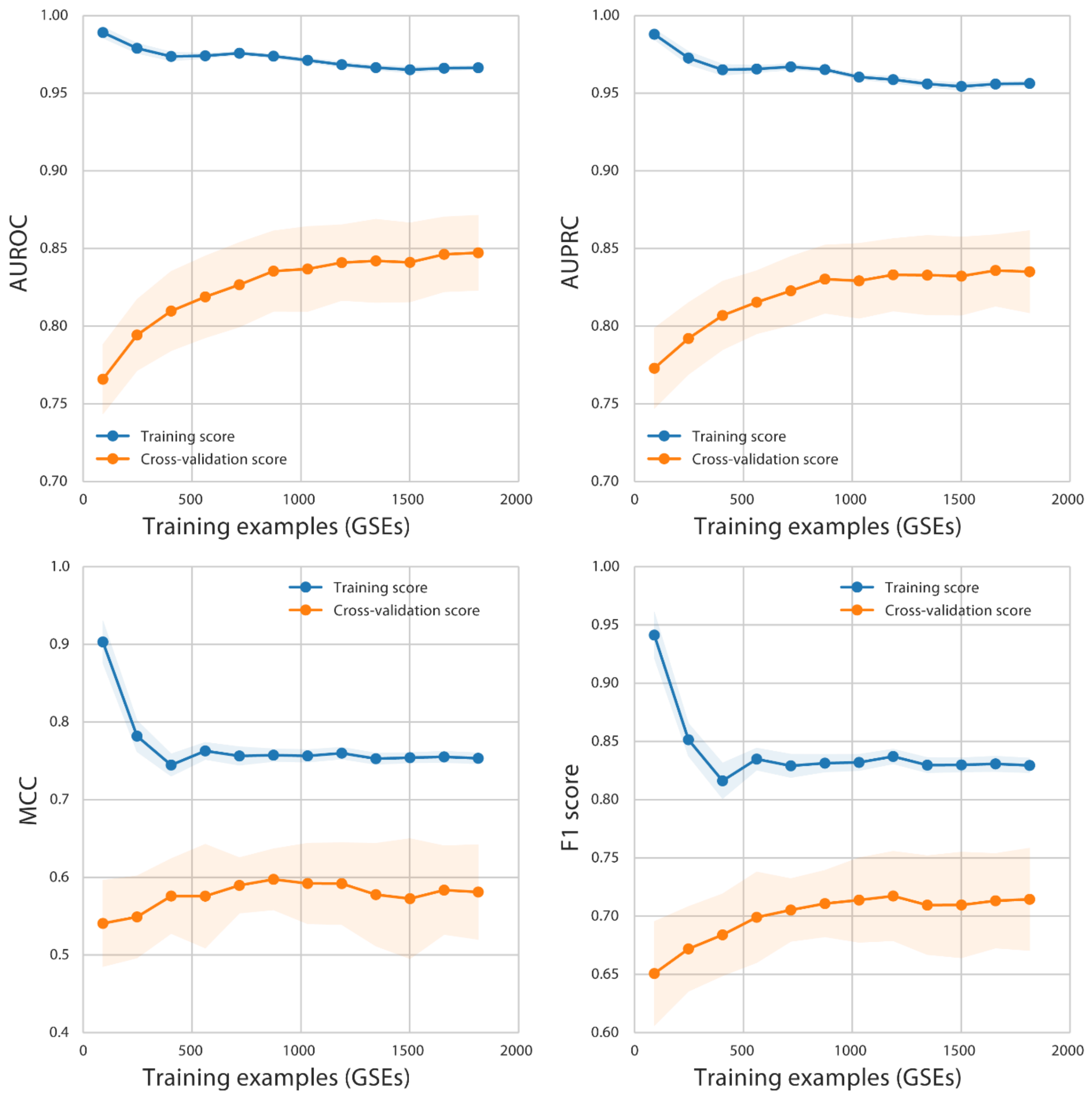

Supplementary figure 10. Learning curves of classification models predicting whether samples are control or perturbations for the three collections of annotated GEO studies.

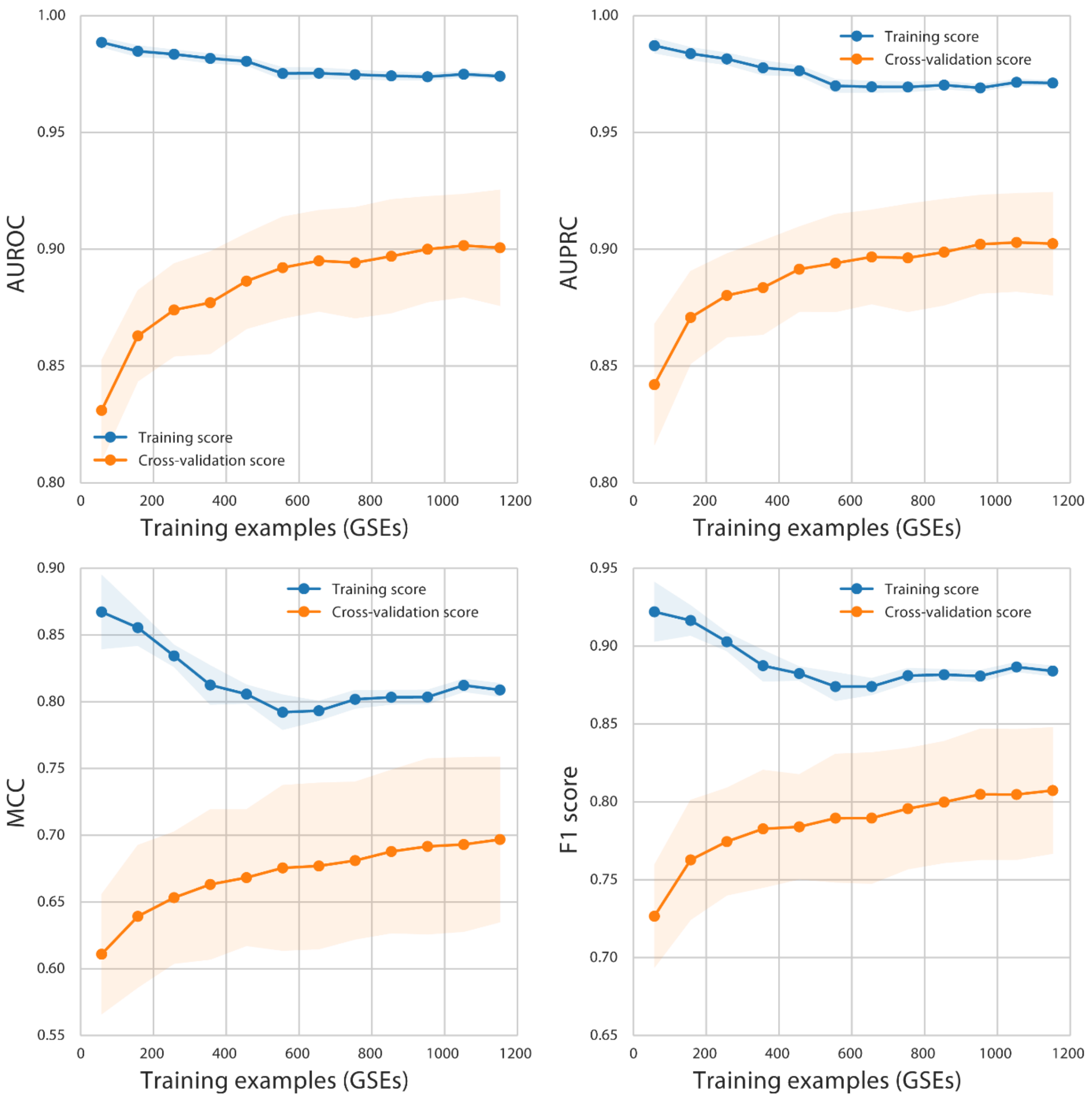

Supplementary figure 11. Learning curves of classification models predicting whether samples are control or perturbations for annotated single-gene perturbation.

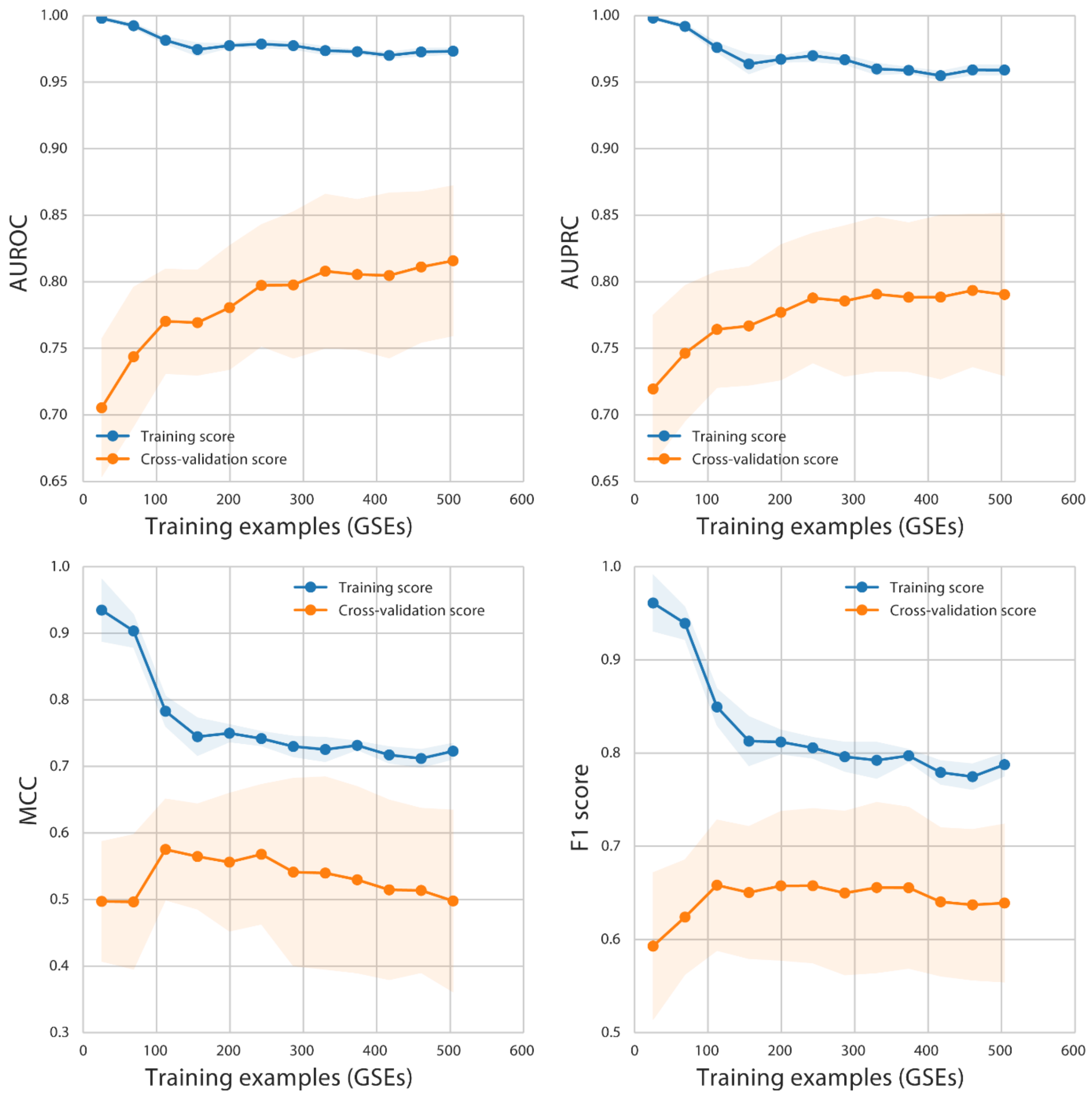

Supplementary figure 12. Learning curves of classification models predicting whether samples are control or perturbations for annotated GEO studies that generate disease signatures.

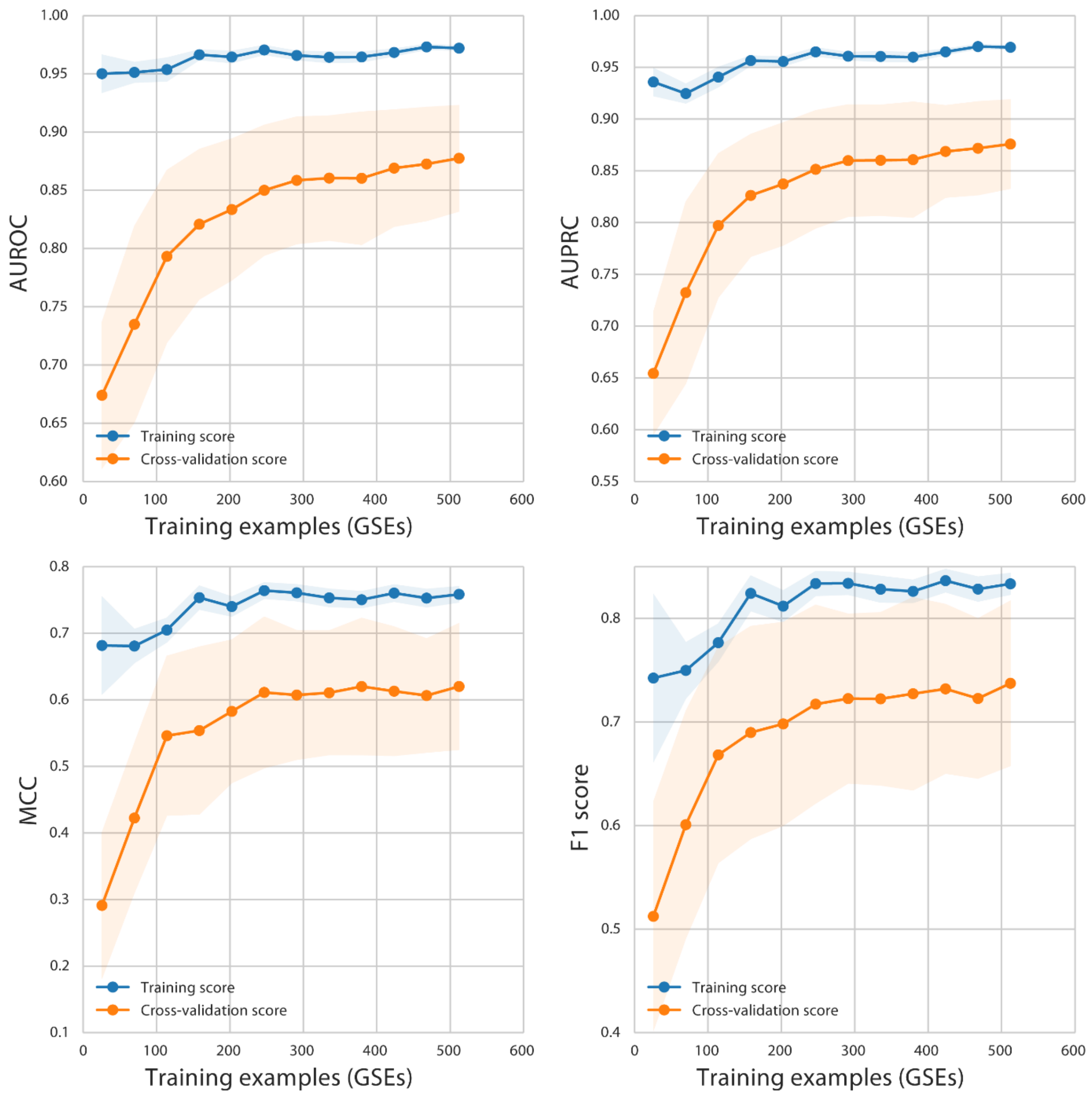

Supplementary figure 13. Learning curves of classification models predicting whether samples are control or perturbations for annotated single-drug perturbation.

Supplementary table 1. Details about matched signatures from MSigDB and CREEDS that do not have significant overlap

CREEDS ID	GEO ID	Control IDs	Perturbation IDs	MSigDB Standard Name	MSigDB Exact Source	Number of Genes in MSigDB	p-value	Reason
gene:1317	GSE6078	GSM140792 GSM140816	GSM140813 GSM140817 GSM140818	HE_PTEN_TARGETS_UP	Fig 2e: red in Mut	15	1	Authors only showed the subset of genes that are related to cell-cycle
gene:1317	GSE6078	GSM140792 GSM140816	GSM140813 GSM140817 GSM140818	HE_PTEN_TARGETS_DN	Fig 2e: green in Mut	7	0.105	Authors only showed the subset of genes that are related to cell-cycle
gene:2842	GSE15102	GSM377671 GSM377675	GSM377676 GSM377677 GSM377678	SAGIV_CD24_TARGETS_UP	Table 1B	22	0.407	Authors showed genes differentially expessed in both siRNA and mAb induction
gene:2842	GSE15102	GSM377671 GSM377675	GSM377676 GSM377677 GSM377678	SAGIV_CD24_TARGETS_DN	Table 1A	45	0.627	Authors showed genes differentially expessed in both siRNA and mAb induction
gene:754	GSE4356	GSM99043 GSM99044 GSM99045	GSM99058 GSM99059 GSM99060	SOUCEK_MYC_TARGETS	Fig. 1aS	7	1	MSigDB curation error: curated genes do not match original figure

Supplementary table 2. Pairs of most similar gene perturbation signatures

Rank	gse_i	pert_type_i	hs_gene_symbol_i	cell_i	gse_j	pert_type_j	hs_gene_symbol_j	cell_j	score	known connection
1	GSE6614	KO	CHRNA4	whole brain	GSE5320	Deficiency	CHRNB4	Brain	1	TRUE
2	GSE4065	Mutation - R225Q	PRKAG3	Skeletal muscle (gastrocnemius)	GSE4067	Mutation (R225Q)	PRKAG3	Skeletal muscle	0.766	TRUE
3	GSE6397	H222P mutation	LMNA	heart	GSE8000	KO	LMNA	whole heart	0.466	TRUE
4	GSE1472	Deficiency	DMD	Leg muscle	GSE1025	Deficiency	DMD	Hindlimb muscle	0.435	TRUE
5	GSE1025	Deficiency	DMD	Hindlimb muscle	GSE1026	Deficiency	DMD	Diaphram	0.429	TRUE
6	GSE897	Deficiency	DMD	Quadriceps	GSE1025	Deficiency	DMD	Hindlimb muscle	0.416	TRUE
7	GSE466	KO	DMD	muscle	GSE1025	Deficiency	DMD	Hindlimb muscle	0.415	TRUE
8	GSE2527	KD	GATA1	megakaryocytes	GSE2433	Truncated Expression	GATA1	Fetal liver	0.411	TRUE
9	GSE34388	KO	DES	young skeletal muscles	GSE41363	KO	DES	tibialis anterior	0.408	TRUE
10	GSE41363	KO	DES	tibialis anterior	GSE34388	depletion	DES	tibialis anterior muscle	0.399	TRUE

11	GSE1472	Deficiency	DMD	Leg muscle	GSE466	KO	DMD	muscle	0.393	TRUE
12	GSE2152	mutation	FH	Uterine fibroid tissue	GSE2724	Heterozygous germline mutations	FH	uterine fibroid and Myometrium	0.377	TRUE
13	GSE897	Deficiency	DMD	Gastroc	GSE1025	Deficiency	DMD	Hindlimb muscle	0.375	TRUE
14	GSE2527	KD	GATA1	megakaryocytes	GSE2433	DeltaN mutation	GATA1	megacariocytes	0.371	TRUE
15	GSE28025	KO	MYBL1	Testis from 17 day old	GSE27568	KO	UBB	Testis	0.369	FALSE
16	GSE1472	Deficiency	DMD	Leg muscle	GSE897	Deficiency	DMD	Gastroc	0.366	TRUE
17	GSE466	KO	DMD	muscle	GSE1025	Deficiency	DMD	Hindlimb muscle	0.363	TRUE
18	GSE466	KO	DMD	muscle	GSE1026	Deficiency	DMD	Diaphram	0.355	TRUE
19	GSE466	KO	DMD	muscle	GSE897	Deficiency	DMD	Quadriceps	0.351	TRUE
20	GSE1472	Deficiency	DMD	Leg muscle	GSE897	Deficiency	DMD	Quadriceps	0.350	TRUE
21	GSE27261	KO	DMRT1	P28 testis tissue	GSE27568	KO	UBB	Testis	0.349	FALSE
22	GSE466	KO	DMD	muscle	GSE897	Deficiency	DMD	Gastroc	0.346	TRUE
23	GSE897	Deficiency	DMD	Extensor digitorum longus	GSE1025	Deficiency	DMD	Hindlimb muscle	0.343	TRUE
24	GSE1025	Deficiency	DMD	Hindlimb muscle	GSE1026	Deficiency	DMD	Diaphram	0.340	TRUE
25	GSE4065	Mutation - R225Q	PRKAG3	Skeletal muscle (gastrocnemius)	GSE4063	KO	PRKAG3	Skeletal muscle	-0.340	TRUE
26	GSE4063	KO	PRKAG3	Skeletal muscle	GSE4067	Mutation (R225Q)	PRKAG3	Skeletal muscle	-0.338	TRUE
27	GSE897	Deficiency	DMD	Quadriceps	GSE1025	Deficiency	DMD	Hindlimb muscle	0.337	TRUE
28	GSE466	KO	DMD	muscle	GSE1025	Deficiency	DMD	Hindlimb muscle	0.336	TRUE
29	GSE9038	CAG knock in	HTT	cerebellum	GSE19780	knock-in	HTT	cerebellum	0.334	TRUE
30	GSE466	KO	DMD	muscle	GSE897	Deficiency	DMD	Extensor digitorum longus	0.333	TRUE
31	GSE897	Deficiency	DMD	Soleus	GSE1026	Deficiency	DMD	Diaphram	0.333	TRUE
32	GSE1025	Deficiency	DMD	Hindlimb muscle	GSE1026	Deficiency	DMD	Diaphram	0.332	TRUE
33	GSE20325	OE	CTNNB1	Embryonic kidney	GSE9629	KO	CTNNB1	Kidney	0.330	TRUE
34	GSE466	KO	DMD	muscle	GSE1026	Deficiency	DMD	Diaphram	0.328	TRUE
35	GSE3889	mutation	SCD	liver	GSE39621	KO	NPC1	liver	0.326	FALSE
36	GSE466	KO	DMD	muscle	GSE897	Deficiency	DMD	Quadriceps	0.324	TRUE
37	GSE28025	KO	MYBL1	Testis from 17 day old	GSE27568	KO	UBB	Testis	0.323	FALSE
38	GSE1472	Deficiency	DMD	Leg muscle	GSE1025	Deficiency	DMD	Hindlimb muscle	0.322	TRUE
39	GSE897	Deficiency	DMD	Quadriceps	GSE1025	Deficiency	DMD	Hindlimb muscle	0.319	TRUE

40	GSE65624	KO	INSR	Liver tissue	GSE3129	Snell mutant PIT1-dw	POU1F1	liver	-0.317	FALSE
41	GSE1472	Deficiency	DMD	Leg muscle	GSE897	Deficiency	DMD	Quadriceps	0.316	TRUE
42	GSE1472	Deficiency	DMD	Leg muscle	GSE1026	Deficiency	DMD	Diaphram	0.316	TRUE
43	GSE1025	Deficiency	DMD	Hindlimb muscle - 56 Days	GSE1026	Deficiency	DMD	Diaphram	0.315	TRUE
44	GSE466	KO	DMD	muscle	GSE1026	Deficiency	DMD	Diaphram	0.315	TRUE
45	GSE466	KO	DMD	muscle	GSE897	Deficiency	DMD	Extensor digitorum longus	0.315	TRUE
46	GSE1472	Deficiency	DMD	Leg muscle	GSE1026	Deficiency	DMD	Diaphram	0.311	TRUE
47	GSE6078	KO	PTEN	Analysis of intestinal polyps	GSE22020	KO	AP1B1	small intestine, epithelial cell	0.310	FALSE
48	GSE897	Deficiency	DMD	Gastroc (mdx mice)	GSE1025	Deficiency	DMD	Hindlimb muscle	0.305	TRUE
49	GSE1472	Deficiency	DMD	Leg muscle	GSE1025	Deficiency	DMD	Hindlimb muscle	0.304	TRUE
50	GSE12049	Deficiency	LAMA2	Hind limb skeletal muscle	GSE3252	Deficiency	LAMA2	Diaphragm	0.302	TRUE

Supplementary table 3. Pairs of most similar disease vs. normal tissue signatures

Rank	gse_i	name_i	cell_i	platform_i	gse_j	name_j	cell_j	platform_j	score	known connection
1	GSE16677	Down syndrome	megakaryocytic leukemia blasts	GPL570	GSE19681	Down syndrome	leukemic blasts	GPL570	0.485	TRUE
2	GSE4130	Dehydration	Hypothalamus	GPL1355	GSE3110	Dehydration	Hypothalamus	GPL1355	0.481	TRUE
3	GSE6798	polycystic ovary syndrome	Skeletal muscle	GPL570	GSE8157	polycystic ovary syndrome	Skeletal muscle	GPL570	0.450	TRUE
4	GSE19429	myelodysplastic syndrome	CD34+ hematopoietic stem cells	GPL570	GSE4619	anemia	CD34+ cells	GPL570	0.437	FALSE
5	GSE19429	myelodysplastic syndrome	CD34+ hematopoietic stem cells	GPL570	GSE4619	anemia	CD34+ cells	GPL570	0.427	FALSE
6	GSE1685	Fracture of femur	Femur	GPL85	GSE1371	Fracture of femur	Femur	GPL85	0.402	TRUE
7	GSE466	Duchenne muscular dystrophy	Muscle - Striated (Skeletal)	GPL81	GSE1472	Duchenne muscular dystrophy	Muscle - Striated (Skeletal)	GPL32	0.393	TRUE
8	GSE19429	myelodysplastic syndrome	CD34+ hematopoietic stem cells	GPL570	GSE4619	anemia	CD34+ cells	GPL570	0.389	FALSE
9	GSE19429	myelodysplastic syndrome	CD34+ hematopoietic stem cells	GPL570	GSE4619	anemia	CD34+ cells	GPL570	0.356	FALSE
10	GSE2527	thrombocytopenia	Megakaryocyte	GPL1261	GSE2433	megakaryocytic leukemia	Megakaryocyte	GPL1261	0.354	FALSE
11	GSE1685	Fracture of femur	Femur	GPL85	GSE594	Bone fracture	Femur	GPL85	0.343	FALSE
12	GSE594	Bone fracture	Femur	GPL85	GSE1371	Fracture of femur	Femur	GPL85	0.342	FALSE
13	GSE19780	Huntington's disease	cerebellum	GPL1261	GSE9038	Huntington's disease	cerebellum	GPL1261	0.334	TRUE

14	GSE4619	MDS - Myelodysplastic syndrome	Bone marrow stem cell	GPL570	GSE19429	myelodysplastic syndrome	CD34+ hematopoietic stem cells	GPL570	0.325	TRUE
15	GSE775	acute myocardial infarction	Heart left ventricles above LAD artery	GPL81	GSE1621	Cardiac Hypertrophy	Myocardial tissue	GPL81	0.313	FALSE
16	GSE1551	dermatomyositis	Muscle - Striated (Skeletal)	GPL96	GSE3112	Polymyositis	Muscle tissue	GPL96	0.311	FALSE
17	GSE4619	MDS - Myelodysplastic syndrome	Bone marrow stem cell	GPL570	GSE19429	myelodysplastic syndrome	CD34+ hematopoietic stem cells	GPL570	0.307	TRUE
18	GSE2724	uterine fibroid	Uterus	GPL96	GSE2725	uterine fibroid	Uterus	GPL96	0.301	TRUE
19	GSE775	acute myocardial infarction	Heart left ventricles above LAD artery	GPL81	GSE1472	Duchenne muscular dystrophy	Muscle - Striated (Skeletal)	GPL32	0.295	FALSE
20	GSE3889	Hypercholesteremia	Hepatic Tissue	GPL1261	GSE2127	hepatocellular carcinoma	Hepatic Tissue	GPL339	0.294	FALSE
21	GSE7999	Tachycardia	Myocardial tissue	GPL1355	GSE4105	myocardial infarction	Myocardial tissue	GPL341	0.294	FALSE
22	GSE34619	Barrett's esophagus	Esopahgus endoscopic biopsies	GPL6244	GSE13083	Barrett's esophagus	Esophageal squamous epithelium	GPL96	0.294	TRUE
23	GSE34619	Barrett's esophagus	esophagus	GPL6244	GSE13083	Barrett's esophagus	Esophageal squamous epithelium	GPL96	0.294	TRUE
24	GSE775	acute myocardial infarction	Heart left ventricles above LAD artery	GPL81	GSE466	Duchenne muscular dystrophy	Muscle - Striated (Skeletal)	GPL81	0.287	FALSE
25	GSE4619	MDS - Myelodysplastic syndrome	Bone marrow stem cell	GPL570	GSE19429	myelodysplastic syndrome	CD34+ hematopoietic stem cells	GPL570	0.286	TRUE
26	GSE17612	schizophrenia	Anterior prefrontal cortex	GPL570	GSE21935	schizophrenia	Superior temporal cortex	GPL570	0.281	TRUE
27	GSE1379	breast cancer	Mammary Gland Tissue	GPL1223	GSE1378	breast cancer	Mammary Gland Tissue	GPL1223	0.281	TRUE
28	GSE1025	Duchenne muscular dystrophy	Muscle - Striated (Skeletal)	GPL81	GSE1472	Duchenne muscular dystrophy	Muscle - Striated (Skeletal)	GPL32	0.280	TRUE
29	GSE4619	MDS - Myelodysplastic syndrome	Bone marrow stem cell	GPL570	GSE19429	myelodysplastic syndrome	CD34+ hematopoietic stem cells	GPL570	0.280	TRUE
30	GSE4619	anemia	CD34+ cells	GPL570	GSE19429	myelodysplastic syndrome	HSC cells	GPL570	0.278	FALSE
31	GSE19780	Huntington's disease	striatum	GPL1261	GSE9038	Huntington's disease	Striatum	GPL1261	0.274	TRUE
32	GSE5389	bipolar disorder	frontal cortex	GPL96	GSE5392	bipolar disorder	brain tissue (dorsolateral prefrontal cortex and orbitofrontal cortex)	GPL96	0.273	TRUE
33	GSE19429	myelodysplastic syndrome	CD34+ hematopoietic stem cells	GPL570	GSE4619	anemia	CD34+ cells	GPL570	0.268	FALSE
34	GSE1871	Acute Lung Injury	Lung Tissue	GPL1261	GSE2640	pulmonary fibrosis	Lung Tissue	GPL339	0.267	FALSE
35	GSE466	Duchenne muscular dystrophy	Muscle - Striated (Skeletal)	GPL81	GSE1026	Duchenne muscular dystrophy	Muscle - Striated (Skeletal) – Diaphragm	GPL81	0.265	TRUE
36	GSE1025	Duchenne muscular dystrophy	Muscle - Striated (Skeletal)	GPL81	GSE466	Duchenne muscular dystrophy	Muscle - Striated (Skeletal)	GPL81	0.264	TRUE

37	GSE1025	Duchenne muscular dystrophy	Muscle - Striated (Skeletal)	GPL81	GSE1026	Duchenne muscular dystrophy	Muscle - Striated (Skeletal) - Diaphragm	GPL81	0.251	TRUE
38	GSE4619	MDS - Myelodysplastic syndrome	Bone marrow stem cell	GPL570	GSE19429	myelodysplastic syndrome	HSC cells	GPL570	0.248	TRUE
39	GSE1472	Duchenne muscular dystrophy	Muscle - Striated (Skeletal)	GPL32	GSE1026	Duchenne muscular dystrophy	Muscle - Striated (Skeletal) – Diaphragm	GPL81	0.247	TRUE
40	GSE13355	Psoriasis vulgaris	Skin tissue	GPL570	GSE14905	Psoriasis vulgaris	Skin tissue	GPL570	0.247	TRUE
41	GSE3112	Polymyositis	Muscle tissue	GPL96	GSE6011	Duchenne muscular dystrophy	Quadriceps	GPL96	0.247	FALSE
42	GSE19429	myelodysplastic syndrome	CD34+ hematopoietic stem cells	GPL570	GSE4619	anemia	CD34+ cells	GPL570	0.245	FALSE
43	GSE775	acute myocardial infarction	Heart left ventricles above LAD artery	GPL81	GSE4710	Heart Injury	Myocardial tissue	GPL339	0.244	FALSE
44	GSE1156	Generalized seizures	Hippocampus	GPL85	GSE4236	status epilepticus	CNS - Brain – Hippocampus	GPL85	0.243	FALSE
45	GSE6710	Psoriasis vulgaris	Skin tissue	GPL96	GSE2503	skin squamous cell carcinoma	Normal skin and squamous cell carcinoma (SCC) tumor	GPL96	0.242	FALSE
46	GSE1551	dermatomyositis	Muscle - Striated (Skeletal)	GPL96	GSE11971	childhood type dermatomyositis	Skeletal muscle biopsies	GPL96	0.240	TRUE
47	GSE4290	oligodendroglioma	CNS - Brain	GPL570	GSE15824	oligodendroglioma	Brain tumor tissue samples of human gliomas	GPL570	0.239	TRUE
48	GSE8000	Emery-Dreifuss muscular dystrophy	whole heart	GPL1261	GSE4710	Heart Injury	Myocardial tissue	GPL339	0.237	FALSE
49	GSE775	acute myocardial infarction	Heart left ventricles above LAD artery	GPL81	GSE1026	Duchenne muscular dystrophy	Muscle - Striated (Skeletal) – Diaphragm	GPL81	0.236	FALSE
50	GSE6710	Psoriasis vulgaris	Skin tissue	GPL96	GSE6475	acne	Skin	GPL571	0.235	FALSE

Supplementary table 4. Pairs of most similar drug perturbation signatures

Rank	gse_i	name_i	cell_i	platform_i	gse_j	name_j	cell_j	platform_j	score	Tanimoto coefficient
1	GSE1839	Diethylstilbestrol	uterus	GPL81	GSE2195	Estradiol	Uterus	GPL81	0.482	0.141
2	GSE46924	Estradiol	MCF-7	GPL570	GSE8597	Estradiol	NA	GPL570	0.418	1.000
3	GSE51207	Cobalt dichloride hexahydrate	H4IIEC3 liver cell line	GPL1355	GSE31503	NICKEL CHLORIDE	H4-II-E-C3 LIVER-derived cell line	GPL1355	0.354	0.000
4	GSE51207	Cobalt dichloride hexahydrate	H4IIEC3 liver cell line	GPL1355	GSE31503	NICKEL CHLORIDE	H4-II-E-C3 LIVER-derived cell line	GPL1355	0.285	0.000
5	GSE12211	imatinib (glivec)	Philadelphia chromosome positive CML CD34+ cells	GPL571	GSE29828	EPZ004777	MV4 -11 leukemia cell line	GPL570	0.265	0.177
6	GSE1839	Diethylstilbestrol	Uterus	GPL81	GSE2195	Estradiol	Uterus	GPL81	0.264	0.141
7	GSE1839	Diethylstilbestrol	Uterus	GPL81	GSE2195	Estradiol	Uterus	GPL81	0.246	0.141
8	GSE12211	imatinib (glivec)	Philadelphia chromosome positive CML CD34+ cells	GPL571	GSE23743	Imatinib	NA	GPL571	0.245	0.915

9	GSE5080	Harman	Astrocytes	GPL570	GSE5023	CYFLUTHRIN	primary fetal ASTROCYTES	GPL570	0.242	0.175
10	GSE53394	Estradiol	MCF-7	GPL96	GSE4025	Tamoxifen	MCF-7	GPL96	0.238	0.070
11	GSE17624	Bisphenol A	Ishikawa endometrial cells	GPL570	GSE8588	PBDE 47	Adrenocortical carcinoma cell line	GPL570	0.234	0.175
12	GSE1922	Imatinib	K562 leukemia cell line	GPL96	GSE19567	Nilotinib	K562 cells	GPL571	0.231	0.510
13	GSE4668	Estradiol	NA	GPL96	GSE4025	Tamoxifen	MCF-7	GPL96	0.229	0.070
14	GSE54711	PLX4032	WM164 BRAF mutant Melanoma cells	GPL6244	GSE50649	PLX4032	COLO829 human melanoma cell line	GPL6244	0.229	1.000
15	GSE19286	Captopril	NA	GPL1261	GSE17297	PRISTANE	Mesentery - DBA mice	GPL1261	0.225	0.048
16	GSE35011	Rosiglitazone	stromal-vascular cells	GPL8321	GSE7111	resveratrol	3T3-L1 progenitor adipocytes	GPL1261	0.224	0.154
17	GSE43695	Bleomycin	LUNG	GPL6246	GSE25640	Bleomycin	Lung	GPL1261	0.223	1.000
18	GSE29828	EPZ004777	MV4 -11 leukemia cell line	GPL570	GSE24493	Imatinib	NA	GPL570	0.221	0.174
19	GSE1922	Imatinib	K562 leukemia cell line	GPL96	GSE19567	Imatinib	K562 cells	GPL571	0.218	1.000
20	GSE53394	Estradiol	MCF-7	GPL96	GSE11352	Estradiol	NA	GPL570	0.217	1.000
21	GSE17624	Bisphenol A	Ishikawa endometrial cells	GPL570	GSE8588	PBDE 47	Adrenocortical carcinoma cell line	GPL570	0.217	0.175
22	GSE15129	ubiquinol	Brain	GPL1261	GSE11291	resveratrol	neocortex	GPL1261	0.217	0.052
23	GSE23743	Imatinib	NA	GPL571	GSE24493	Imatinib	NA	GPL570	0.215	1.000
24	GSE35011	Rosiglitazone	stromal-vascular cells	GPL8321	GSE17297	PRISTANE	Mesentery - DBA mice	GPL1261	0.215	0.034
25	GSE17297	PRISTANE	Mesentery	GPL1261	GSE42813	Vitamin E	aorta	GPL1261	0.215	0.467
26	GSE4025	Tamoxifen	NA	GPL96	GSE4668	Estradiol	MCF7/BUS human breast cancer cells	GPL96	0.214	0.070
27	GSE46924	Estradiol	MCF-7 cell line	GPL570	GSE26834	Estradiol	MCF-7 breast cancer cells	GPL571	0.213	1.000
28	GSE2640	Bleomycin	NA	GPL339	GSE2565	Phosgene	Lungs	GPL339	0.213	0.011
29	GSE35011	Rosiglitazone	NA	GPL8321	GSE21329	troglitazone	adipose tissue	GPL341	0.211	0.398
30	GSE8597	Estradiol	NA	GPL570	GSE26834	Estradiol	MCF-7 breast cancer cells	GPL571	0.207	1.000
31	GSE1458	rosiglitazone	3T3-L1 adipocytes	GPL81	GSE35011	Rosiglitazone	NA	GPL8321	0.207	1.000
32	GSE53394	Estradiol	MCF-7	GPL96	GSE4668	Estradiol	MCF7/BUS human breast cancer cells	GPL96	0.203	1.000
33	GSE1922	Imatinib	K562 leukemia cell line	GPL96	GSE19567	Nilotinib	K562 cells	GPL571	0.201	0.510
34	GSE4025	Tamoxifen	NA	GPL96	GSE4668	Estradiol	MCF7/BUS human breast cancer cells	GPL96	0.201	0.070
35	GSE1922	Imatinib	K562 leukemia cell line	GPL96	GSE19567	Nilotinib	K562 cells	GPL571	0.199	0.510

36	GSE1922	Imatinib	K562 leukemia cell line	GPL96	GSE19567	Nilotinib	K562 cells	GPL571	0.199	0.510
37	GSE35011	Rosiglitazone	NA	GPL8321	GSE21329	Pioglitazone	adipose	GPL341	0.199	0.600
38	GSE53394	Estradiol	MCF-7 breast cancer (BC) cells	GPL96	GSE4668	Estradiol	NA	GPL96	0.199	1.000
39	GSE17297	PRISTANE	Mesentery - DBA mice - Day 3	GPL1261	GSE15129	coenzyme Q10	liver	GPL1261	0.198	0.072
40	GSE11670	Deferasirox	K562 (human myeloid leukemia cells)	GPL570	GSE24493	Imatinib	NA	GPL570	0.198	0.197
41	GSE1922	Imatinib	K562 leukemia cell line (VIII)	GPL96	GSE19567	Nilotinib	K562 cells	GPL571	0.197	0.510
42	GSE11670	Deferasirox	K562 (human myeloid leukemia cells)	GPL570	GSE8565	Argyrin A	MCF7 breast adenocarcinoma cells	GPL570	0.197	0.143
43	GSE43695	Bleomycin	Lung (Fra-1 deficient mutants)	GPL6246	GSE25640	Bleomycin	Lung	GPL1261	0.196	1.000
44	GSE12211	imatinib (glivec)	Philadelphia chromosome positive CML CD34+ cells	GPL571	GSE6930	Doxorubicin	A673	GPL4685	0.194	0.131
45	GSE1922	Imatinib	K562 leukemia cell line (VIII)	GPL96	GSE19567	Imatinib	K562 cells	GPL571	0.193	1.000
46	GSE1922	Imatinib	K562 leukemia cell line	GPL96	GSE19567	Imatinib	K562 cells	GPL571	0.192	1.000
47	GSE37441	Vemurafenib	SK-MEL-28 melanoma cell line (vemurafenib sensitive cell line) - 6 Hours	GPL10558	GSE24862	PLX4032	M249 melanoma cell line	GPL6244	0.192	1.000
48	GSE11670	Deferasirox	K562 (human myeloid leukemia cells)	GPL570	GSE8565	Argyrin A	MCF7 breast adenocarcinoma cells	GPL570	0.192	0.143
49	GSE54711	PLX4032	WM164 BRAF mutant Melanoma cells	GPL6244	GSE50649	PLX4032	COLO829 human melanoma cell line-MITF	GPL6244	0.192	1.000
50	GSE29828	EPZ004777	MV4 -11 leukemia cell line - Day 6	GPL570	GSE23743	Imatinib	NA	GPL571	0.191	0.174

Supplementary table 5. Tables of top-matched drugs and potential predicted mimickers

Drug	geo_id	pert_id	pert_desc	pubchem_id	score
cycloheximide	GSE8597	BRD-A62184259	NA	2900	0.4988
		BRD-K36055864	NA	6197	0.5747
		BRD-K06792661	Narciclasine	72376	0.5811
		BRD-K91370081	NA	253602	0.5873
		BRD-A62184259	Cycloheximide	2900	0.6061
		BRD-A62184259	NA	2900	0.6089
		BRD-K03067624	EMETINE HYDROCHLORIDE	10219	0.6115
		BRD-K80348542	NA	442195	0.6198
		BRD-A24643465	homoharringtonine	16219462	0.6237
		BRD-A62184259	Cycloheximide	2900	0.6273
		BRD-K36055864	CYCLOHEXIMIDE	6197	0.6288
		BRD-A25687296	NA	5702048	0.6306
		BRD-A62184259	NA	2900	0.6311
		BRD-K80348542	NA	442195	0.6364
		BRD-A62184259	NA	2900	0.6375
		BRD-A62184259	Cycloheximide	2900	0.6376
		BRD-K36055864	CYCLOHEXIMIDE	6197	0.6416
		BRD-K76674262	NA	285033	0.6458
		BRD-A62184259	Cycloheximide	2900	0.6464
		BRD-A62184259	Cycloheximide	2900	0.6479
Azacitidine	GSE29077	BRD-K03406345	azacitidine	9444	0.8068
		BRD-K15563106	NA	4788	0.8098
		BRD-K64642496	NA	10322450	0.8114
		BRD-K44100512	KIN001-043	NA	0.8129
		BRD-K68336408	Tyrphostin AG 1478	2051	0.8145
		BRD-K01121114	AT-MLPCN CSC-006	49843203	0.8172
		BRD-A17065207	Brefeldin A	5362868	0.8187
		BRD-A02481876	Importazole	2949965	0.8207
		BRD-K03406345	azacitidine	9444	0.8208
		BRD-K44100512	KIN001-043	NA	0.8222
		BRD-A47829399	artesunate	73707396	0.826
		BRD-K23984367	sorafenib	406563	0.8279
		BRD-K39503511	MK-0591	60923	0.828
		BRD-K97330509	Src Kinase Inhibitor II	1172104	0.8303
		BRD-K03816923	Rottlerin	5281847	0.8305
		BRD-K03406345	azacitidine	9444	0.8308
		BRD-A31107743	89671	5353446	0.8332
		BRD-K03816923	Rottlerin	5281847	0.8332
		BRD-K78513633	Lonidamine	39562	0.8335

		BRD-K21672174	Ro 28-1675 ?	9886086	0.8337
Tretinoin	GSE1588	BRD-K44100512	KIN001-043	NA	0.8703
		BRD-K06854232	NA	2126	0.8998
		BRD-K71879491	tretinoin	444795	0.9015
		BRD-K49685476	GR-105	5289501	0.9019
		BRD-K74980345	NA	56643211	0.9048
		BRD-K44100512	KIN001-043	NA	0.9052
		BRD-K62493605	JAS07_009	24747231	0.9052
		BRD-K44100512	KIN001-043	NA	0.906
		BRD-K90699611	Acitretin	5284513	0.9067
		BRD-K06854232	AM580	2126	0.9081
		BRD-K76723084	isotretinoin	5282379	0.9093
		BRD-K71879491	tretinoin	444795	0.9096
		BRD-K16189898	CHIR-99021	9956119	0.9098
		BRD-K08547377	irinotecan hcl)trihydrate)	23581792	0.9098
		BRD-K49685476	GR-105	5289501	0.9106
		BRD-K31342827	GF 109203X	2396	0.9114
		BRD-K35483542	GR-101	449171	0.912
		BRD-K68246049	NA	5354022	0.912
		BRD-K44100512	KIN001-043	NA	0.9123
		BRD-K31342827	GF-109203X	2396	0.9128
Vemurafenib	GSE37441	BRD-K03618428	PP-110	24905203	0.517
		BRD-K03449891	foretinib	42642645	0.5288
		BRD-K03449891	foretinib	42642645	0.5297
		BRD-K49865102	PD-0325901	9826528	0.5349
		BRD-U51024685	HG-6-64-01	NA	0.5379
		BRD-K16478699	PLX-4720	24180719	0.5404
		BRD-K16478699	PLX-4720	24180719	0.5411
		BRD-K56343971	vemurafenib	42611257	0.5417
		BRD-U60236422	WH-4-025	NA	0.5418
		BRD-K03449891	foretinib	42642645	0.542
		BRD-K41895714	AS605240	10377751	0.5435
		BRD-K20696416	NVP-AEW541	NA	0.5437
		BRD-K95435023	PHA-665752	10461815	0.5456
		BRD-K50140147	NVP-TAE684	16038120	0.546
		BRD-K49865102	PD-0325901	9826528	0.5464
		BRD-K98490050	NA	3926765	0.5467
		BRD-K49865102	PD-0325901	9826528	0.5471
		BRD-A58767537	afatinib	22225683	0.5474
		BRD-K05104363	PD-184352	6918454	0.548
		BRD-K24496482	SB590885	NA	0.5485

Dexamethasone	GSE34313	BRD-A69951442	dexamethasone	73707402	0.7743
		BRD-K46056750	AZD-7762	67077825	0.7814
		BRD-A79768653	sirolimus	5374464	0.7864
		BRD-A93255169	THALIDOMIDE	5426	0.7882
		BRD-A40639672	KETOROLAC TROMETHAMINE	3826	0.7903
		BRD-K24675965	LY 288513	2802894	0.7916
		BRD-A92177080	BETAMETHASONE ACETATE	45006158	0.7935
		BRD-K49328571	dasatinib	3062316	0.7938
		BRD-K30697463	desoximetasone	5311067	0.7942
		BRD-K23875128	Rho kinase inhibitor III [rockout]	644354	0.7947
		BRD-A46186775	HYDROCORTISONE PHOSPHATE TRIETHYLAMINE	6602442	0.7955
		BRD-K32164935	TOLAZAMIDE	5503	0.7969
		BRD-K62310379	fluticasone	444036	0.7982
		BRD-A35108200	Dexamethasone	3003	0.8009
		BRD-A93255169	THALIDOMIDE	5426	0.8016
		BRD-A42628519	IOPANIC ACID	3735	0.8031
		BRD-K35240538	methylprednisolone	6741	0.8033
		BRD-A02180903	BETAMETHASONE	6710614	0.8047
		BRD-A15297126	FLUOCINONIDE	6710662	0.8065
		BRD-K23875128	Rho kinase inhibitor III [rockout]	644354	0.8066
Dexamethasone	GSE7683	BRD-A01346607	FLUMETHASONE	5702178	0.8649
		BRD-K61480498	GR-231	3247059	0.8665
		BRD-A10188456	NA	5702035	0.8706
		BRD-A36010170	NA	5702266	0.8758
		BRD-A69951442	dexamethasone	73707402	0.8779
		BRD-K30697463	desoximetasone	5311067	0.8787
		BRD-K71035033	S1064	10074640	0.8788
		BRD-A67862938	NA	312915	0.8794
		BRD-A27887842	NA	5702106	0.8805
		BRD-A49765801	NA	5702172	0.8811
		BRD-K28470988	L-690,330	132449	0.8829
		BRD-A42628519	NA	3735	0.8839
		BRD-K81709173	NA	443943	0.8849
		BRD-A40639672	NA	3826	0.8853
		BRD-K49865102	PD-0325901	9826528	0.8855
		BRD-A93424738	NA	5702036	0.8857
		BRD-A92439610	TRIAMCINOLONE ACETONIDE	5702126	0.886
		BRD-K69328504	L-690,488	5132514	0.8864
		BRD-K69328504	L-690,488	5132514	0.8865
		BRD-K51556300	NA	44506645	0.8865
Dexamethasone	GSE54608	BRD-A01346607	FLUMETHASONE	5702178	0.8465

		BRD-A42628519	IOPANIC ACID	3735	0.8606
		BRD-A35108200	Dexamethasone	3003	0.8619
		BRD-A35108200	betamethasone	3003	0.8659
		BRD-A02180903	BETAMETHASONE	6710614	0.8661
		BRD-A46186775	HYDROCORTISONE PHOSPHATE TRIETHYLAMINE	6602442	0.8667
		BRD-A49765801	FLURANDRENOLIDE	5702172	0.867
		BRD-A26095496	CLOBETASOL PROPIONATE	5702274	0.8688
		BRD-A66861218	BETAMETHASONE 17,21-DIPROPIONATE	6708733	0.8691
		BRD-K07668032	NCGC00012272-02	3234850	0.8694
		BRD-A82238138	Budesonide	2462	0.871
		BRD-A60571864	BUDESONIDE	5702148	0.8713
		BRD-K29173907	Isoflupredone acetate	224246	0.8715
		BRD-A92177080	BETAMETHASONE ACETATE	45006158	0.8738
		BRD-K24675965	LY 288513	2802894	0.8742
		BRD-A37780065	TRIAMCINOLONE	5702125	0.8745
		BRD-A69951442	dexamethasone	73707402	0.8747
		BRD-A35108200	Dexamethasone	3003	0.8755
		BRD-K28470988	L-690,330	132449	0.8775
		BRD-A15297126	FLUOCINONIDE	6710662	0.8776
Sorafenib	GSE39192	BRD-K87909389	alvocidib	5287969	0.9428
		BRD-K43389698	BMS-387032	3025986	0.9433
		BRD-K79090631	CGP-60474	644215	0.9448
		BRD-K21680192	mitoxantrone	5458171	0.9453
		BRD-K87909389	alvocidib	5287969	0.9459
		BRD-K13390322	AT-7519	11338033	0.9461
		BRD-K23984367	sorafenib	406563	0.9475
		BRD-K87909389	alvocidib	5287969	0.9477
		BRD-K56343971	vemurafenib	42611257	0.9478
		BRD-K87909389	alvocidib	5287969	0.9481
		BRD-K79090631	CGP-60474	644215	0.9487
		BRD-K43389698	BMS-387032	3025986	0.9487
		BRD-K49865102	PD-0325901	9826528	0.949
		BRD-K79090631	CGP-60474	644215	0.9491
		BRD-K19220233	JNK-9L	59588070	0.9493
		BRD-K43389698	BMS-387032	3025986	0.9494
		BRD-K13390322	AT-7519	11338033	0.9494
		BRD-K43389698	BMS-387032	3025986	0.9498
		BRD-K43389698	BMS-387032	3025986	0.9501
		BRD-K56751279	Y-39983	9810884	0.9504
trichostatin A	GSE1437	BRD-K04887706	AKT-inhibitor-1-2	10196499	0.8798
		BRD-K45044657	NA	NA	0.8893

		BRD-K81418486	vorinostat	5311	0.8931
		BRD-A19037878	trichostatin A	6376322	0.8932
		BRD-K77908580	S1053	4261	0.8951
		BRD-K53308430	NA	44507247	0.8961
		BRD-A19037878	trichostatin A	6376322	0.8963
		BRD-K68202742	trichostatin A	444732	0.8978
		BRD-K77908580	NA	4261	0.8984
		BRD-K53308430	NA	44507247	0.8986
		BRD-A39646320	H7270	3571	0.8987
		BRD-K53903639	480743.cdx	19582717	0.8988
		BRD-K77908580	S1053	4261	0.8994
		BRD-A19037878	trichostatin A	6376322	0.9
		BRD-K52522949	NA	11395181	0.9004
		BRD-A42649439	API-2	290486	0.9014
		BRD-K52522949	NA	11395181	0.9014
		BRD-A19037878	trichostatin A	6376322	0.9025
		BRD-K77908580	NA	4261	0.9032
		BRD-K81418486	vorinostat	5311	0.9034
Tretinoin	GSE32161	BRD-K06926592	NA	444795	0.8034
		BRD-K68246049	NA	5354022	0.812
		BRD-K71879491	tretinoin	444795	0.8146
		BRD-K90699611	Acitretin	5284513	0.8171
		BRD-K06854232	AM580	2126	0.8188
		BRD-K06926592	Isotretinoin	444795	0.82
		BRD-A96799240	GR-109	6438629	0.8214
		BRD-K35483542	R4643	449171	0.8236
		BRD-K71879491	tretinoin	444795	0.8333
		BRD-K06854232	NA	2126	0.8401
		BRD-K62012036	GR-108	5284513	0.8406
		BRD-K06854232	NA	2126	0.8476
		BRD-K06926592	Isotretinoin	444795	0.8503
		BRD-K51290057	NA	6184667	0.8544
		BRD-A17718497	NA	46912148	0.8591
		BRD-K76723084	isotretinoin	5282379	0.8617
		BRD-K49685476	GR-105	5289501	0.8628
		BRD-K35483542	GR-101	449171	0.8629
		BRD-K49685476	GR-105	5289501	0.8652
		BRD-K78844995	NA	127898	0.8681
Methylprednisolone	GSE490	BRD-A92439610	TRIAMCINOLONE ACETONIDE	5702126	0.8619
		BRD-K35240538	methylprednisolone	6741	0.8657
		BRD-K20696416	NVP-AEW541	NA	0.8661

		BRD-K31627533	NA	5311412	0.8666
		BRD-K70771662	11K-629S	1471787	0.8683
		BRD-A07000685	HYDROCORTISONE HEMISUCCINATE	5702069	0.8695
		BRD-K16533489	NA	23891056	0.8702
		BRD-A65767837	HYDROCORTISONE ACETATE	5702068	0.8705
		BRD-A25687296	NA	5702048	0.8706
		BRD-A98283014	C3930	644274	0.8707
		BRD-A13133631	FLUOROMETHOLONE	5702058	0.8731
		BRD-A75409952	wortmannin	5691	0.8732
		BRD-K40175214	torin-1	49836027	0.8746
		BRD-U25771771	NA	NA	0.8748
		BRD-A26199074	2561	2531	0.8764
		BRD-K04853698	LDN-193189	25195294	0.8766
		BRD-K30697463	desoximetasone	5311067	0.877
		BRD-K23875128	Rho kinase inhibitor III [rockout]	644354	0.8771
		BRD-K63770300	NCGC00188740-01	56643207	0.8771
		BRD-K35960502	NICLOSAMIDE	4477	0.8773
Doxorubicin	GSE58074	BRD-K92093830	doxorubicin	443939	0.7096
		BRD-K03829970	NA	53338854	0.7132
		BRD-K92093830	Doxorubicin hydrochloride	443939	0.7232
		BRD-K21680192	mitoxantrone	5458171	0.7291
		BRD-U64521890	XMD16-144	NA	0.7311
		BRD-K04548931	epirubicin	65348	0.7327
		BRD-K21680192	mitoxantrone	5458171	0.7335
		BRD-K11927976	NA	9799509	0.7364
		BRD-K64890080	BI 2536	11364421	0.7385
		BRD-K21680192	mitoxantrone	5458171	0.7385
		BRD-A13122391	16-HYDROXYTRIPTOLIDE	16220015	0.7455
		BRD-K83794624	P8624	11296583	0.7464
		BRD-K83794624	P8624	11296583	0.7465
		BRD-K19724398	NA	53338857	0.7475
		BRD-K13514097	S1120	6442177	0.7481
		BRD-K04548931	NA	65348	0.7503
		BRD-K21680192	mitoxantrone	5458171	0.7506
		BRD-K21680192	mitoxantrone	5458171	0.7511
		BRD-U64521890	XMD16-144	NA	0.754
		BRD-K21680192	mitoxantrone	5458171	0.7547
Lapatinib	GSE38376	BRD-K03618428	PP-110	24905203	0.7653
		BRD-K69932463	AZD8055	25262965	0.7709
		BRD-K19687926	lapatinib	208908	0.7758
		BRD-A75409952	wortmannin	5691	0.7828

		BRD-K34581968	BMS-536924	11353973	0.7833
		BRD-K03618428	PP-110	24905203	0.7839
		BRD-A18328003	GDC-0980	NA	0.7843
		BRD-K12184916	NA	11977753	0.7845
		BRD-A18328003	GDC-0980	NA	0.7845
		BRD-A58767537	afatinib	22225683	0.7848
		BRD-A75409952	wortmannin	5691	0.785
		BRD-K67868012	PI-103	9884685	0.7853
		BRD-K52911425	GDC-0941	17755052	0.7857
		BRD-K08799216	pelitinib	6445562	0.7859
		BRD-K67566344	KU-0063794	16736978	0.786
		BRD-K69932463	AZD8055	25262965	0.7866
		BRD-K03618428	PP-110	24905203	0.7874
		BRD-K08799216	pelitinib	6445562	0.7885
		BRD-A18328003	GDC-0980	NA	0.7886
		BRD-K12994359	Valdecoxib	119607	0.7888
